# GPC6 facilitates progression of SHH-subgroup medulloblastoma by enhancing Hedgehog secretion and signaling responses

**DOI:** 10.7555/JBR.39.20250406

**Published:** 2026-05-21

**Authors:** Yue Wang, Qingyue Meng, Qin Zhu, Xinyi Zhang, Xinfa Wang, Junping He, Jing Cai, Xiaohong Pu, Zihe Ai, Qinya Li, Kedui Pu, Tingting Yu, Chen Liu, Shen Yue

**Affiliations:** 1 Department of Medical Genetics, Nanjing Medical University, Nanjing, Jiangsu 211166, China; 2 Institute of Geriatric Medicine, Jiangsu Province Geriatric Hospital, Nanjing, Jiangsu 210009, China; 3 Department of Neurosurgery, Children's Hospital of Nanjing Medical University, Nanjing, Jiangsu 210093, China; 4 Department of Pathology, Nanjing Drum Tower Hospital, the Affiliated Hospital of Nanjing University Medical School, Nanjing, Jiangsu 210008, China

**Keywords:** glypican-6, SHH signaling, medulloblastoma, extracellular vesicles, primary cilia

## Abstract

Medulloblastoma (MB) is the most common malignant tumor of the cerebellum in children. The Sonic Hedgehog (SHH) subgroup of MB (SHH-MB) is driven by aberrant activation of the SHH pathway; however, mutations in genes related to this pathway are relatively rare, posing challenges for therapeutic development. Glypican-6 (GPC6), a heparan sulfate proteoglycan, is highly expressed in SHH-MB. In this study, we demonstrate the synchronous expression of GPC6 with GLI family zinc finger 1 (GLI1) in both the developing cerebellum and medulloblastoma. GPC6 promotes cell proliferation, migration, and invasion in SHH-MB cell lines (DAOY and ONS-76). Consistently, GPC6 enhances the SHH pathway activity by upregulating GLI1 expression, supports ciliogenesis essential for signal transduction, and facilitates SHH ligand expression *via* extracellular vesicles. These findings suggest that GPC6 acts as a key regulator of SHH signaling and represents a potential therapeutic target in SHH-MB.

## Introduction

Medulloblastoma (MB) is the most common malignant tumor of the cerebellum in children, originating from neural stem or progenitor cells^[1]^. MB typically arises in the midline of the vermis, often extending into the fourth ventricle or spreading along the subarachnoid space and spinal axis^[2–3]^. Because of its high genetic heterogeneity, MB is classified into four molecular subgroups: Wingless-related integration site (WNT), Sonic Hedgehog (SHH), Group 3, and Group 4^[4–5]^. Among these, the SHH subgroup (SHH-MB) accounts for approximately one-third of all cases and is characterized by aberrant activation of the SHH signaling pathway^[6]^.

In the SHH signaling pathway, the binding of the SHH ligand to the patched 1 (PTCH1) receptor alleviates its repression of the downstream G protein-coupled receptor Smoothened (SMO). This activation triggers the GLI transcription factors, which in turn initiate the transcription of SHH target genes^[7]^. Previous studies have shown that somatic mutations in SHH pathway genes, including *PTCH1* and *SMO*, are frequently associated with the formation of SHH-MB. Nevertheless, the overall incidence of somatic mutations in SHH-MB is lower than that in other solid tumors^[8]^. The limited number of mutations, combined with an incomplete understanding of MB development and progression, poses significant challenges for the development of effective targeted therapies.

The SHH signaling pathway relies not only on intracellular signal transduction within the responding cells but also on the secretion and extracellular transport of the SHH ligand. During SHH signal biogenesis, the ligand undergoes covalent modification with two lipids, cholesterol and palmitate, which are essential for its activity^[9–12]^. Dispatched homolog 1 (DISP1) facilitates the release of the dually lipid-modified SHH signal from the membrane of SHH-producing cells and, together with its carrier protein, signal peptide, CUB domain and EGF-like domain-containing protein 2 (SCUBE2), generates a soluble morphogen^[13]^. Once released, SHH, with its lipid adducts, is transferred *via* its co-receptors to PTCH1, triggering downstream signaling in responding cells. Although signal transduction in responding cells has been extensively characterized, the mechanisms governing SHH secretion and transport remain less well understood^[14]^.

As key components of the extracellular matrix, glycoproteins are crucial for modulating tumor cell responses to their microenvironment. Glypicans (GPCs), belonging to the heparan sulfate proteoglycan (HSPG) family, are anchored to the outer surface of the plasma membrane *via* a glycosylphosphatidylinositol (GPI) anchor. To date, six members of the GPC family (GPC1–6) have been identified in mammals, which are classified into two subfamilies: GPC3/5 and GPC1/2/4/6^[15]^. Structurally, GPCs are highly conserved and share common features, including a 60–70 kDa core protein, 14 conserved cysteine residues, 2–4 long linear glycosaminoglycan (GAG) chains, and a C-terminal GPI anchor^[16–17]^. The GPI anchor can be cleaved by the lipase NOTUM, releasing GPCs from the cell surface, and most GPCs can also be processed by furin-like convertases, resulting in truncated forms of 30–40 kDa^[15–16]^. GPCs regulate the activity of various signaling molecules, including SHH, fibroblast growth factor (FGF), WNT, and bone morphogenetic proteins (BMPs)^[16]^.

Notably, GPCs have conserved orthologs in *Drosophila melanogaster*, namely Dally (corresponding to GPC3/5) and Dally-like protein, Dlp (corresponding to GPC1/4/6)^[16]^. Both Dally and Dlp have been implicated as co-receptors of the Hedgehog ligand and play essential roles in mediating both short-range and long-range Hedgehog signaling during development. Similar to mammalian GPCs, their activity is regulated by Notum, which cleaves the GPI anchor and modulates their association with the cell membrane^[18]^. These conserved structural and functional features suggest an evolutionarily preserved role of glypicans in fine-tuning Hedgehog signaling.

Among the six GPC family members, GPC6 is one of the most abundant extracellular matrix molecules in the brain, playing a critical role in nervous system development and regulation^[19–20]^. GPC6 is essential for embryonic morphogenesis. Biallelic mutations in *GPC6* lead to omodysplasia (OMOD1, MIM 258315), a condition marked by severe short stature, limb shortening, facial dysmorphisms, and developmental delay due to reduced Hedgehog signaling in the developing long bones^[21–22]^. Additionally, GPC6 has been reported to promote embryonic stomach growth and intestinal elongation by enhancing Hedgehog signaling^[23–24]^.

These observations raise an important question regarding whether GPC6 functionally contributes to SHH-subgroup medulloblastoma and how it may regulate SHH pathway activity. Therefore, this study aimed to define the role of GPC6 in SHH-MB and to elucidate the mechanisms by which it influences Hedgehog signal reception and ligand distribution. To achieve this, we integrated analyses of patient tissues with gain- and loss-of-function experiments in SHH-MB cell lines, CRISPR-edited models, as well as ciliary imaging and extracellular vesicle assays. Through these approaches, our work was designed to establish a mechanistic framework for understanding the contribution of GPC6 to SHH-MB biology.

## Materials and methods

### Animals

All animal procedures were approved by the Animal Ethical and Welfare Committee of Nanjing Medical University (IACUC-14030113-4) and conducted in compliance with the guidelines for the care and use of laboratory animals. Female *Ptch*^+/−^ mice (JAX stock #003081, Jackson Laboratory, Bar Harbor, ME, USA) were housed and maintained under specific pathogen-free conditions in the animal facility of Nanjing Medical University.

### Cells, plasmids, and siRNAs

Human cell lines were acquired from the following sources: the American Type Culture Collection (ATCC; Manassas, VA, USA), FuHeng Cell Center (Shanghai, China), and Procell Life Science & Technology (Wuhan, China). Wild-type (WT), *Sufu*^−/−^, and *Ptch1*^−/− ^mouse embryonic fibroblasts (MEFs) were previously described^[25]^. The cells used in this study included DAOY cells (ATCC, Cat. #HTB-186, RRID: CVCL_1167), cultured in DMEM medium (Wisent, Saint-Jean-Baptiste, QC, Canada), ONS-76 cells (RRID: CVCL_1624), maintained in RPMI-1640 medium (Wisent), hTERT-RPE1 cells (ATCC, Cat. #CRL-4000, RRID: CVCL_4388), grown in DMEM/F12 media (Wisent), MEF cells (ATCC, Cat. #CRL-2991, RRID: CVCL_L690), cultured in DMEM medium (Wisent), and 293T cells (ATCC, Cat. #CRL-3216, RRID: CVCL_0063), cultured in DMEM medium (Wisent). All cells were cultured in medium supplemented with 10% fetal bovine serum (FBS, Wisent), 1× glutamine (Gibco, Grand Island, NY, USA), 1× sodium pyruvate (Gibco), and 1% penicillin/streptomycin (Thermo Fisher Scientific, Waltham, MA, USA) under standard conditions (37 ℃, 5% CO_2_)^[26]^.

Full-length human *GPC6* cDNA was produced using PCR and subsequently cloned into the pcDNA3.1 (RRID: Addgene_128034) and pCDH-CMV vector (RRID: Addgene_72265), with an HA tag appended at the C-terminus. *SHH*-N cDNA was also generated by PCR and inserted into the pRK5 vector (RRID: Addgene_32693). *GPC6*ΔGPI was created by amplifying amino acids 1–529 from the *GPC6* full-length clone and adding an HA tag at the C-terminus. siRNAs specific for human *GPC1*–*6* and *GLI1* were procured from GenePharma (Shanghai, China).

### Cell transfection and treatment

Cells were transfected with the indicated plasmids using NEOFECT DNA transfection reagent (TF201201, Neofect Beijing Biotech) or with siRNAs using JetPRIME transfection reagent (CPT-114, Polyplus) according to the manufacturers' protocols. Forty-eight hours after transfection, cells were treated with GDC-0449 (100 μmol/L; S1082, Selleck) or homemade SHH-conditioned medium (SHH-CM; 1∶10 dilution) in DMEM supplemented with 0.5% FBS for 24 h^[25]^.

### Lentiviral production

All plasmids used in the current study were kindly provided by Professor Jingjing Ben (Nanjing Medical University). To package lentiviral particles, 293T cells were seeded in 10 cm dishes and transfected with either the pCDH-*GPC6*-T2A-copGFP or pCDH-*GPC6*ΔGPI-T2A-copGFP plasmids, following the standard protocol. After 48 h of culture, the lentivirus-enriched medium was collected, filtered through a 0.45 μm pore-sized filter (Millipore, Bedford, MA, USA), and stored at −80 ℃ for future use.

### CRISPR-Cas9 genome editing

Genome editing in DAOY cells was performed using the CRISPR-Cas9 technique. Two single-guide RNAs (sgRNAs) specific to the first exon of the human *GPC6* gene were designed and subsequently inserted into the pX330 vector (RRID: Addgene_101733). The targeting sequences for sgRNAs are shown in ***Supplementary Table 1***. CRISPR/Cas9 plasmids and empty plasmids carrying puromycin resistance genes were co-transfected into the cells. Forty-eight hours post-transfection, the cells were treated with puromycin (2 μg/mL) for 4 days and then cultured for an additional 3 days without selection to facilitate expansion. Surviving DAOY cells were plated at a density of one cell per well in 96-well plates to generate single-cell colonies. These colonies were subsequently screened by genotyping and validated by Western blotting (WB) analysis.

### Extracellular vesicle (EV) isolation and characterization

EVs were isolated from 293T cell-conditioned medium using a differential ultracentrifugation protocol^[27]^. The conditioned medium was collected daily for three consecutive days post-transfection. It underwent a series of centrifugation steps: 300 *g* for 10 min to remove dead cells, 2000 *g* for 10 min to remove cellular debris, 12000 *g* for 70 min to remove apoptotic bodies and microvesicles, and 100000 *g* for 70 min to concentrate EVs. These steps were performed using an Optima XPN-100 Ultracentrifuge (Beckman Coulter, Brea, CA, USA). The final pellets were resuspended in sterile phosphate-buffered saline (PBS) and stored at −80 ℃ for further analysis. The size distribution and particle concentration of EVs were quantified using nanoparticle tracking analysis (NTA) on a ZetaView® analyzer (Particle Metrix GmbH, Meerbusch, Germany) using ZetaView software (v8.06.01 SP1), following the manufacturer's protocols. For structural validation, EVs were adsorbed onto carbon-coated copper grids, negatively stained with 2% uranyl acetate (for 1.5 min), and imaged using a Tecnai G2 Spirit BioTWIN transmission electron microscope (FEI Company, Hillsboro, OR, USA). Images were acquired with an Olympus iTEM camera system (Olympus Soft Imaging Solutions GmbH, Münster, Germany).

### Indirect co-culture using a two-chamber system

The co-culture of ONS-76 cells and 293T cells transfected with *SHH*-N and *GPC6* plasmids was conducted using a two-chamber transwell system^[28]^. To investigate the proliferation of ONS-76 cells in co-culture with 293T cells, transfected 293T cells were seeded onto 0.4 µm-pore polycarbonate membrane inserts (Millipore, Bedford, MA, USA), while ONS-76 cells were placed at the bottom of a six-well plate. Co-cultured cells were maintained for 48 h in DMEM containing 5% FBS, allowing for the exchange of secreted vesicles and soluble factors. Proliferation was assessed using 5-ethynyl-2'-deoxyuridine (EdU) staining with the EdU Cell Proliferation Assay Kit (Beyotime, Shanghai, China). Images were captured using a fluorescence microscope, and proliferating cells were quantified in five randomly selected fields. Additionally, a modified transwell assay was used to assess migration and invasion. Briefly, transfected 293T cells were plated in the lower chamber, while ONS-76 cells were seeded in the upper chamber. Migration and invasion assays, as mentioned above, were performed in DMEM supplemented with 0.5% FBS to minimize serum-induced effects.

### Immunohistochemical (IHC) staining

MB microtissue arrays were obtained from Nanjing Drum Tower Hospital. Fresh MB tissue samples were collected from the Children's Hospital of Nanjing Medical University, with written informed consent provided by all participating patients or their legal guardians prior to tissue collection. This study was conducted in accordance with the ethical principles of the Declaration of Helsinki and was approved by the Institutional Review Boards of the Children's Hospital of Nanjing Medical University and Nanjing Drum Tower Hospital.

The cerebella from wild-type postnatal mice at various developmental ages, as well as spontaneous MB from *Ptch*^+/−^ mice, were dissected and fixed in 4% paraformaldehyde at 4 ℃ for 24 h, and then paraffin-embedded. Sections were cut serially to a thickness of 5 µm using a Leica microtome (Leica Biosystems, Wetzlar, Germany). IHC staining was performed using a rabbit two-step detection kit (ZSGB-BIO, Beijing, China) according to standard protocols. Briefly, tissues were deparaffinized, rehydrated, subjected to antigen retrieval, and then treated to block endogenous peroxidase activity. The following primary antibodies were used: rabbit anti-GPC6 (1∶100, Cat. #A2741, ABclonal, Wuhan, China) and rabbit anti-GLI1 (1∶50, Cat. #HA500465, HUABIO, Hangzhou, Zhejiang, China). The sections were then incubated with the signal enhancement reagent and subsequently with the horseradish peroxidase (HRP)-conjugated polymer-enhanced goat anti-rabbit IgG secondary antibody from the kit. Finally, chromogenic development, counterstaining, dehydration, and mounting were carried out. For semi-quantitative analysis, an experienced pathologist evaluated the staining intensity (0: none, 1: weak, 2: moderate, 3: strong) and determined the percentage of immunopositive cells according to the following scale: 0 (< 5%), 1 (6%–25%), 2 (26%–50%), 3 (51%–75%), and 4 (> 76%)^[29]^. Final IHC scores were calculated as the product of intensity and percentage scores.

### Hematoxylin and eosin (H&E) staining

For routine histopathological assessment, paraffin-embedded tissue sections (5 µm) were subjected to H&E staining. Briefly, sections were deparaffinized, rehydrated through graded ethanol, and stained with hematoxylin to visualize nuclei. After differentiation and bluing, sections were counterstained with eosin to highlight cytoplasmic and extracellular components. Finally, the slides were dehydrated, cleared, and mounted for microscopic examination.

### Immunofluorescence staining

For primary cilia studies, cells were plated on gelatin-coated coverslips (0.4 × 10^5^ cells per coverslip) and grown to 90% confluence, then serum-starved in DMEM for 24 h. For EV studies, 80 µL of resuspended concentrated EVs were diluted in 420 µL PBS, resulting in a total of 500 µL, and added to a 24-well plate containing a gelatin-coated coverslip. Samples were spinoculated at 1200 *g* at 13 ℃ for 2 h to allow EV attachment to the coverslips^[30]^. Cells or EVs were fixed with 4% paraformaldehyde for 20 min at 4 ℃ and permeabilized with 0.1% Triton X-100 for 10 min. Standard procedures for immunostaining were followed, except for GPC6 and CD63, for which no permeabilization was applied. Cells or EVs were then blocked for 1 h with PBS containing 3% bovine serum albumin, followed by incubation with primary antibodies overnight at 4 ℃: rabbit anti-GPC6 (1∶100, Cat. #A2741, ABclonal), rabbit anti-CD63 (1∶100, Cat. #HA722731, HUABIO), and mouse anti-SHH (1∶500, Cat. #sc-365112, Santa Cruz, Dallas, TX, USA), and then with secondary antibodies for 2 h at room temperature: donkey anti-mouse IgG (H + L) highly cross-adsorbed secondary antibody, Alexa Fluor 488 (1∶200, Cat. #A21202, Thermo Fisher Scientific), and donkey anti-rabbit IgG (H + L) highly cross-adsorbed secondary antibody, Alexa Fluor 594 (1∶200, Cat. #A21207, Thermo Fisher Scientific). Nuclei were counterstained with DAPI. Cells and EVs were visualized using a laser confocal microscope (Zeiss LSM710, Carl Zeiss, Oberkochen, Germany; ANDOR BC43, Oxford Instruments, Belfast, UK).

### WB analysis

Cells, EVs, or tissue homogenates were lysed using a modified RIPA buffer at 4 ℃ for 1 h as previously described^[25]^. Lysates were sonicated for 30 s and clarified by centrifugation at 12000 *g* for 20 min. Protein concentrations were quantified using a bicinchoninic acid (BCA) assay kit (Thermo Fisher Scientific). Equal quantities of protein were mixed with 5× SDS-PAGE sample loading buffer, denatured at 95 ℃ for 5 min, and resolved by SDS-PAGE. Proteins were transferred to PVDF membranes and immunoblotted using the following primary antibodies overnight at 4 ℃: mouse anti-β-actin (1∶5000, Cat. #AF7018, Affinity, Cincinnati, OH, USA), mouse anti-GAPDH (1∶5000, Cat. #AF7021, Proteintech, Wuhan, China), goat anti-GPC6 (1∶1000, Cat. #AF2845, R&D, Minneapolis, MN, USA), rabbit anti-GPC6 (1∶1000, Cat. #A2741, ABclonal), rabbit anti-GLI1 (1∶1000, Cat. #S2534, Cell Signaling Technology, Boston, MA, USA), rabbit anti-HA (1∶1000, Cat. #51064-2-AP, Proteintech), mouse anti-SHH (1∶500, Cat. #sc-365112, Santa Cruz), rabbit anti-CD63 (1∶1000, Cat. #HA722731, HUABIO), rabbit anti-HSP70 (1∶1000, Cat. #ET1601-11, HUABIO), rabbit anti-ALIX (1∶1000, Cat. #ET1705-74, HUABIO), mouse anti-E-cadherin (1∶1000, Cat. #14472, Cell Signaling Technology), rabbit anti-N-cadherin (1∶1000, Cat. #E-AB-15993, Elabscience, Wuhan, China), and rabbit anti-MMP3 (1∶1000, Cat. #17873-1-AP, Proteintech). Then, after three washes with 0.2% Tris-buffered saline with Tween 20 (TBST), the membranes were incubated with the appropriate HRP-conjugated secondary antibody at room temperature for 2 h. The secondary antibodies used were: goat anti-rabbit IgG HRP (1∶5000, Cat. #111-005-003, Jackson ImmunoResearch, West Grove, PA, USA), goat anti-mouse IgG HRP (1∶5000, Cat. #115-035-003, Jackson ImmunoResearch), and rabbit anti-goat IgG HRP (1∶5000, Cat. #305-005-045, Jackson ImmunoResearch). Following additional washes, protein signals were detected using an enhanced chemiluminescence (ECL) detection reagent (Thermo Fisher Scientific).

### Reverse transcription-quantitative PCR (RT-qPCR) analysis

Using RNAiso Plus reagent (TaKaRa, Kyoto, Japan), total RNA was isolated from cultured cells and subsequently reverse transcribed with HiScript ⅡQ RT SuperMix (Cat. #R223-01, Vazyme, Nanjing, Jiangsu, China). Quantitative PCR (qPCR) was performed using AceQ qPCR SYBR Green Master Mix (Cat. #Q111-02, Vazyme). All measurements were performed in triplicate, and β-actin (*ACTB*) served as the internal control for normalization. The qPCR primer sequences are shown in ***Supplementary Table 2.***

### Flow cytometric analysis, cell counting kit-8 (CCK-8), and colony formation assays

DAOY and ONS-76 cells were seeded in 96-well plates at a density of 0.4 × 10^4^ cells per well. After adhesion, cell viability was assessed every 24 h over a 72-h period using the CCK-8 kit (Cat. #A311-02, Vazyme). Specifically, 10 μL of the CCK-8 solution was added to each well containing complete medium. After incubation for 2 h in a cell culture incubator, the absorbance at 450 nm was measured using a microplate reader. The cell viability percentage was calculated according to the following formula: cell viability (%) = [OD (experiment) – OD (blank)]/[OD (control) – OD (blank)] × 100%.

To assess the cell cycle state, cells were seeded in 6-well plates and transfected with siRNA for 48 h. After transfection, the cells were harvested, fixed with pre-chilled 75% ethanol, and then analyzed for cell cycle distribution using a flow cytometer (BD Biosciences, Franklin Lakes, NJ, USA).

For the colony formation assay, transfected cells were seeded in 6-well plates at a density of 200 cells per well in triplicate and cultured for 2 weeks. Then, the cells were fixed using 4% paraformaldehyde at 4 ℃ for 15 min, followed by staining with 0.1% crystal violet for 30 min. Colonies containing more than 50 cells were required for counting.

### Cell migration and invasion assays

Cell migration and invasion were evaluated using Transwell chambers (8 μm pore size; Corning, Corning, NY, USA). For the migration assay, transfected tumor cells (2.5 × 10^4^ cells/well) were suspended in 300 μL of serum-free medium and seeded into the upper chamber. The lower chamber was filled with 600 μL of medium supplemented with 10% FBS. For the invasion assay, membranes were pre-coated with 60 μL of Matrigel (1∶30 dilution in serum-free medium; Corning) to simulate extracellular matrix barriers. Both assays were conducted in a 5% CO_2_ humidified incubator at 37 ℃ for 12 h. According to the experimental design, an SHH-neutralizing antibody 5E1 (1∶500, sc-365112, Santa Cruz) or the SMO antagonist GDC0449 (100 μmol/L, S1082, Selleck) was added to the culture medium to treat the cells. After incubation, migrated or invaded cells on the lower membrane surface were fixed with methanol, stained with 0.1% crystal violet (Beyotime), and quantified by counting five randomly selected fields per insert under an inverted light microscope.

### Analysis of published datasets

Publicly available human medulloblastoma expression datasets GSE85217 and GSE124814 were analyzed to evaluate GPC family expression patterns. Pearson correlation analysis was performed to assess pairwise associations between genes of interest. Heatmaps were generated to visualize relative expression levels of GPCs across samples. The hazard ratio with a 95% confidence interval and the log-rank *P* value were calculated, with statistical significance defined as *P* < 0.05.

### Statistical analysis

All statistical analyses were performed using GraphPad Prism (version 9.5.0). Each experiment was independently repeated at least three times. Statistical comparisons were performed using an unpaired two-tailed Student's *t*-test or one-way/two-way ANOVA followed by an appropriate multiple comparisons test. *P* values < 0.05 were considered statistically significant.

## Results

### Enhanced GPC6 expression in SHH-MB within the GPC family

**Figure 1:**
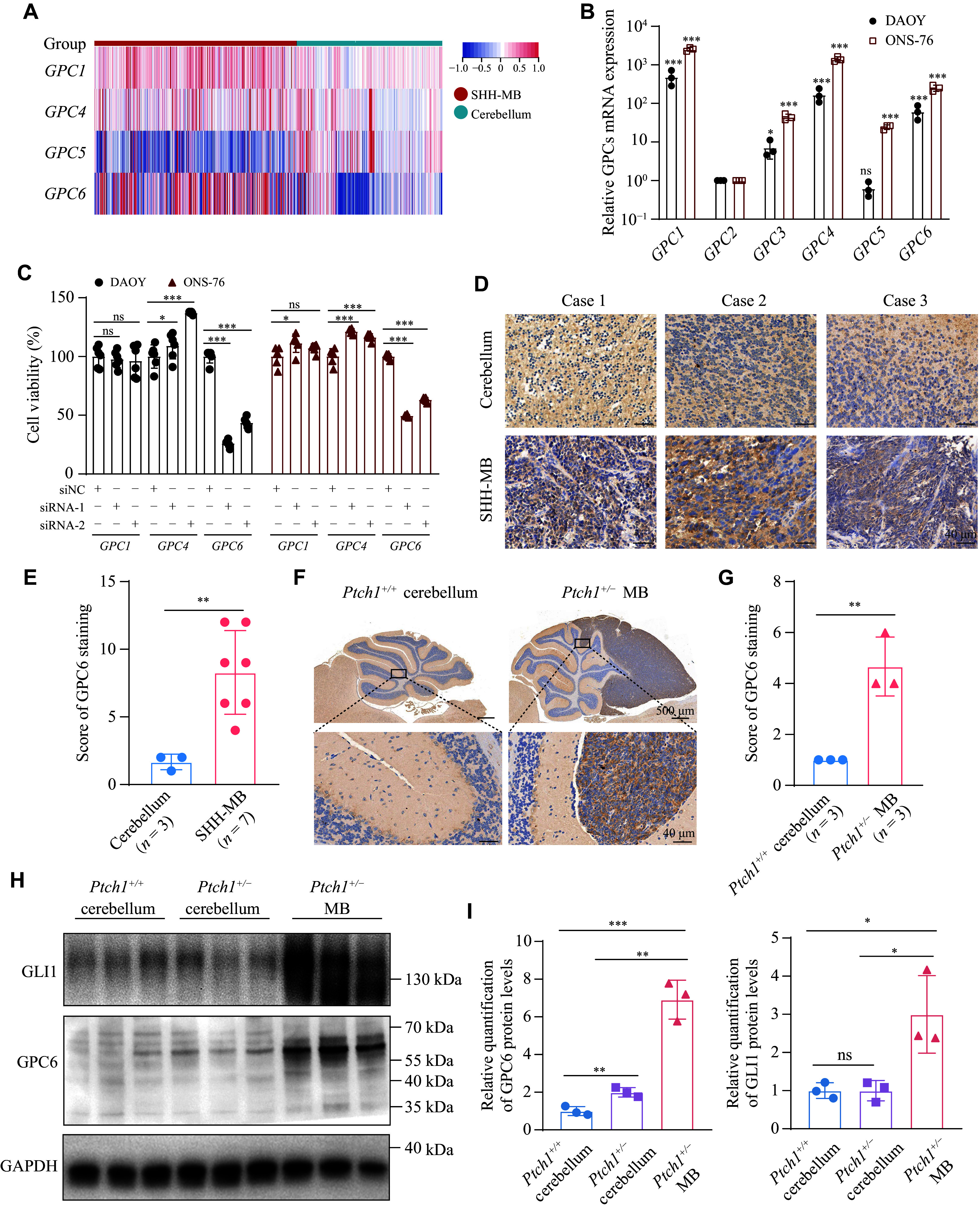
The abundance of GPC6 expression was upregulated in the Sonic Hedgehog (SHH) subgroup of medulloblastoma (MB). A: Comparison of the mRNA abundance of glypicans in normal cerebella (*n* = 291) and the SHH subgroup MB (*n* = 405). Data sourced from the GSE124814 dataset. B: The expression of glypican mRNA in DAOY and ONS-76 cells, standardized by *GPC2*, the least abundantly expressed glypican. C: Cell viability of DAOY and ONS-76 cells transfected with siRNAs (20 nmol/L) targeting individual GPC family members was evaluated using the CCK-8 assay after 72 h of culture. D and E: Representative immunohistochemical (IHC) staining of GPC6 (D) and quantification of GPC6 IHC scores (E) in normal cerebellar tissues (*n* = 3) and SHH subgroup MB tissues (*n* = 7). Scale bar, 40 μm. F and G: Representative IHC staining of GPC6 in normal cerebellum and spontaneous MB of *Ptch1*^+/−^ mice (*n* = 3 per group), and corresponding quantification of GPC6 IHC scores. H and I: Western blotting detection of GPC6 and GLI1 protein levels in the cerebellum of wild-type mice, normal cerebellum, and spontaneous MB from *Ptch1*^+/−^ mice, with corresponding quantification of protein levels shown as mean ± standard error of the mean from three independent samples. ^*^P < 0.05, ^**^P < 0.01, and ^***^P < 0.001; ns represents not significant by one-way ANOVA followed by Dunnett's test compared with *GPC2* (B) or compared with the siNC group (C), Mann-Whitney *U* test (E and G), and one-way ANOVA followed by Tukey's multiple comparisons test (I).

To investigate the potential role of glypicans in SHH-MB, a comparative analysis of the mRNA expression profiles of glypican family members was performed using the GEO dataset GSE124814, which contains 405 SHH-MB and 291 normal cerebellum tissues. Notably, *GPC1*, *GPC4*, and *GPC6* exhibited elevated levels of expression in SHH-MB specimens, while *GPC5* expression was downregulated (***Fig. 1A***). *GPC2* and *GPC3* were not available in the dataset. The expression levels of glypicans also varied across the four different molecular subgroups of MB (***Supplementary Fig. 1A***). For verification, the expression patterns of the GPC family members were examined in multiple MB cell lines, including SHH-MB cell lines DAOY and ONS-76, Group 3 cell line D341, and Group 4 cell line D283 (***Supplementary Fig. 1B***). Consistent with the tissue data, *GPC1*, *GPC4*, and *GPC6* demonstrated notably higher expression levels within the two SHH-MB cell lines compared with other GPC family members (***Fig. 1B***). The impact of glypicans on SHH-MB cell proliferation was assessed by individually silencing each glypican in DAOY cells using small interfering RNAs (siRNAs) against them. Among glypicans, *GPC6* depletion most prominently curtailed the proliferative capacity of both DAOY and ONS-76 cells (***Fig. 1C*** and ***Supplementary Fig. 1C–1E***), without inducing notable changes in the expression levels of other GPC family members, except for an upregulation of *GPC5* (***Supplementary Fig. 1F***). These results establish GPC6 as a promising candidate for further investigation.

IHC staining was performed on clinical SHH-MB samples, revealing increased GPC6 protein expression in MB tissues compared with para-cancerous tissues (***Fig. 1D*** and ***1E***). *Ptch1* heterozygous (*Ptch1*^*+/−*^) mice, which are predisposed to develop medulloblastoma spontaneously and exhibit activation of the SHH downstream transcription factor GLI1, also showed markedly elevated GPC6 expression within *Ptch1*^*+/−*^ MB compared with normal cerebellar tissues, as confirmed by IHC analysis (***Fig. 1F*** and ***1G***). These observations were further supported by WB analysis, demonstrating similar trends (***Fig. 1H*** and ***1I***). In summary, these findings highlight the heightened expression of GPC6, a member of the glypican family, in SHH-MB.

### Synchronous expression of GPC6 with GLI1 in both developing cerebellum and MB

**Figure 2:**
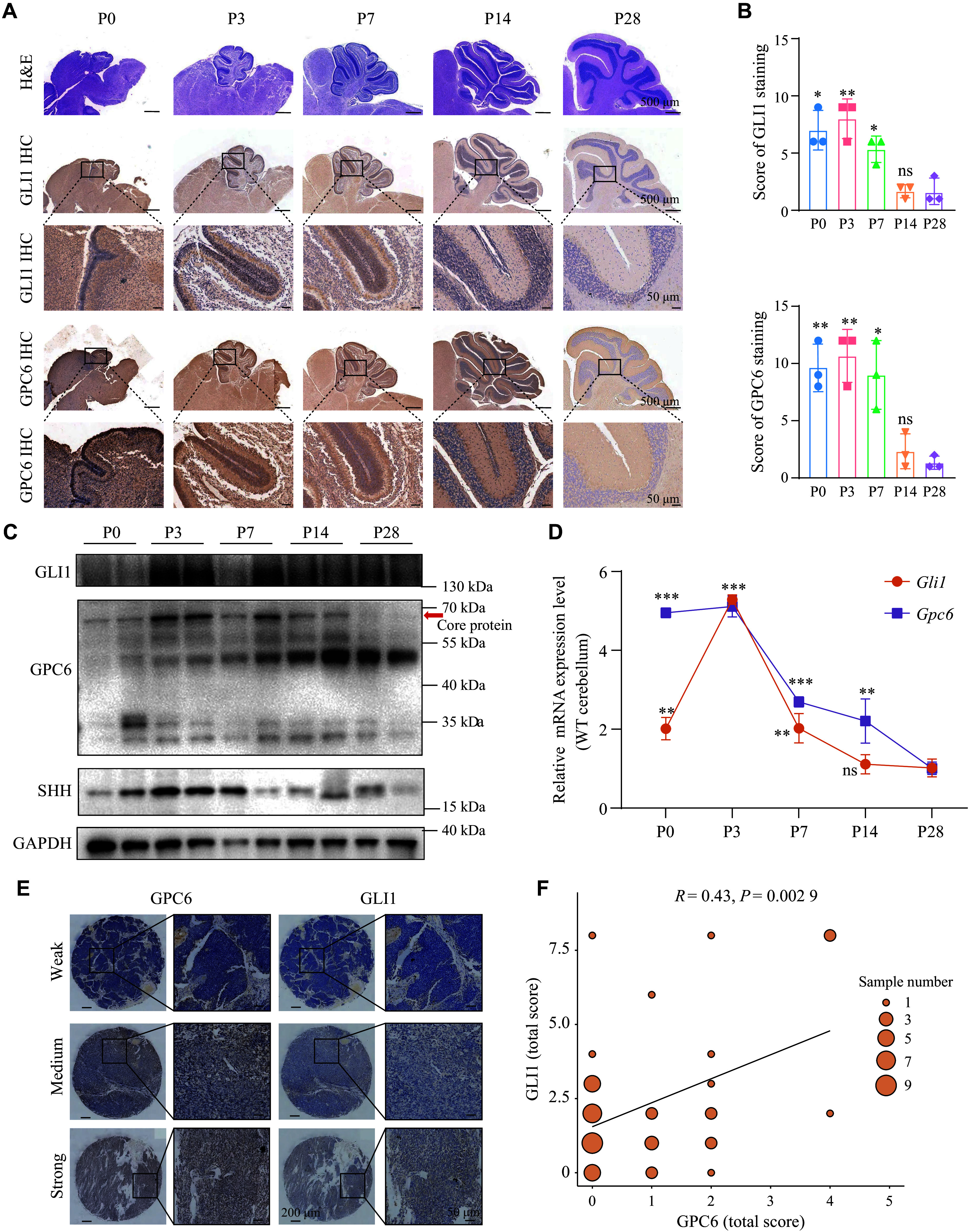
Expression patterns of GPC6 and GLI1 were consistent in developing cerebella and medulloblastoma (MB) tissues. A: Hematoxylin and eosin (H&E) and immunohistochemical (IHC) staining of GPC6 and GLI1 in developing cerebella of wild-type (WT) mice. P0, P3, P7, P14, and P28 denote the indicated postnatal days of mice. Scale bar, 500 μm or 50 μm. B: Quantification of GPC6 and GLI1 IHC scores from three biologically independent samples at each developmental stage. C: Western blotting detection of GLI1, GPC6, and SHH protein levels during cerebellar development in normal mice. Red arrows show the core protein of GPC6. Quantitative analyses of WB data are presented in ***Supplementary Fig. 2A***–***2C***. D: The expression of *Gpc6* and *Gli1* mRNAs in the developing cerebella of WT mice, standardized by P28. E: Representative IHC staining of GPC6 and GLI1 in MB tissue array (*n* = 47). Scale bar, 200 μm (full-section) and 50 μm (magnified region). F: IHC score distribution of GLI1 and GPC6 staining and scatter plot of Pearson's correlation between GLI1 and GPC6 expression. ^*^P < 0.05, ^**^P < 0.01, and ^***^P < 0.001; ns represents not significant by one-way ANOVA followed by Dunnett's test compared with the P28 group (B and D). Abbreviations: GLI1, GLI family zinc finger 1; GPC6, glypican-6; SHH, Sonic Hedgehog.

SHH-MB originates from cerebellar granular neuronal precursors (GNPs) following the aberrant activation of the SHH pathway^[6,31–32]^. During cerebellar development, GNPs proliferate extensively in the external granule layer (EGL) in response to SHH secreted by Purkinje cells^[31,33]^. We performed IHC staining to evaluate the expression of GPC6 and the SHH pathway target GLI1 during postnatal cerebellar development. The results revealed that during the early stage of postnatal cerebellar development, from postnatal day 0 (P0) to P7, GPC6 exhibited its highest expression in the EGL, accompanied by elevated GLI1 expression in that region (***Fig. 2A*** and ***2B***). From P14 to P28, as GNP cells migrated from the EGL to the inner granule layer (IGL) to form mature cerebellar granule neurons, the levels of GLI1 gradually decreased, and GPC6 expression also declined (***Fig. 2A***). The reduction in postnatal GPC6 expression was further confirmed by WB analysis (***Fig. 2C*** and ***Supplementary Fig. 2A***) and RT-qPCR (***Fig. 2D***). This decrease was correlated with the concurrent reduction in SHH and GLI1 levels during murine cerebellar development (***Fig. 2C***, ***2D***, and ***Supplementary Fig. 2B*** and ***2C***). Additionally, an approximately 50 kDa GPC6 isoform was detected in postnatal cerebellar tissues, with antigen competition assays confirming its specificity, suggesting that it may represent a previously uncharacterized GPC6 isoform expressed after P7 (***Fig. 2C*** and ***Supplementary Fig. 2D***).

Elevated levels of GLI1 were observed in clinical MB samples^[34]^. To determine the correlation between GPC6 and GLI1 levels in MB, GPC6 and GLI1 expression levels were examined in a tumor tissue array consisting of 49 unstratified MB specimens. Using a semi-quantitative scoring matrix considering both IHC staining intensity and the percentage of positively stained cells, a moderate yet statistically significant Pearson correlation was clearly present between GPC6 and GLI1 expression (***Fig. 2E*** and ***2F***). The positive correlation between GPC6 and GLI1 levels in the developing cerebella and MBs suggests that GPC6 is involved in SHH signaling and its downstream cell growth.

### GPC6 promoted the growth, migration, and invasion of DAOY cells

**Figure 3:**
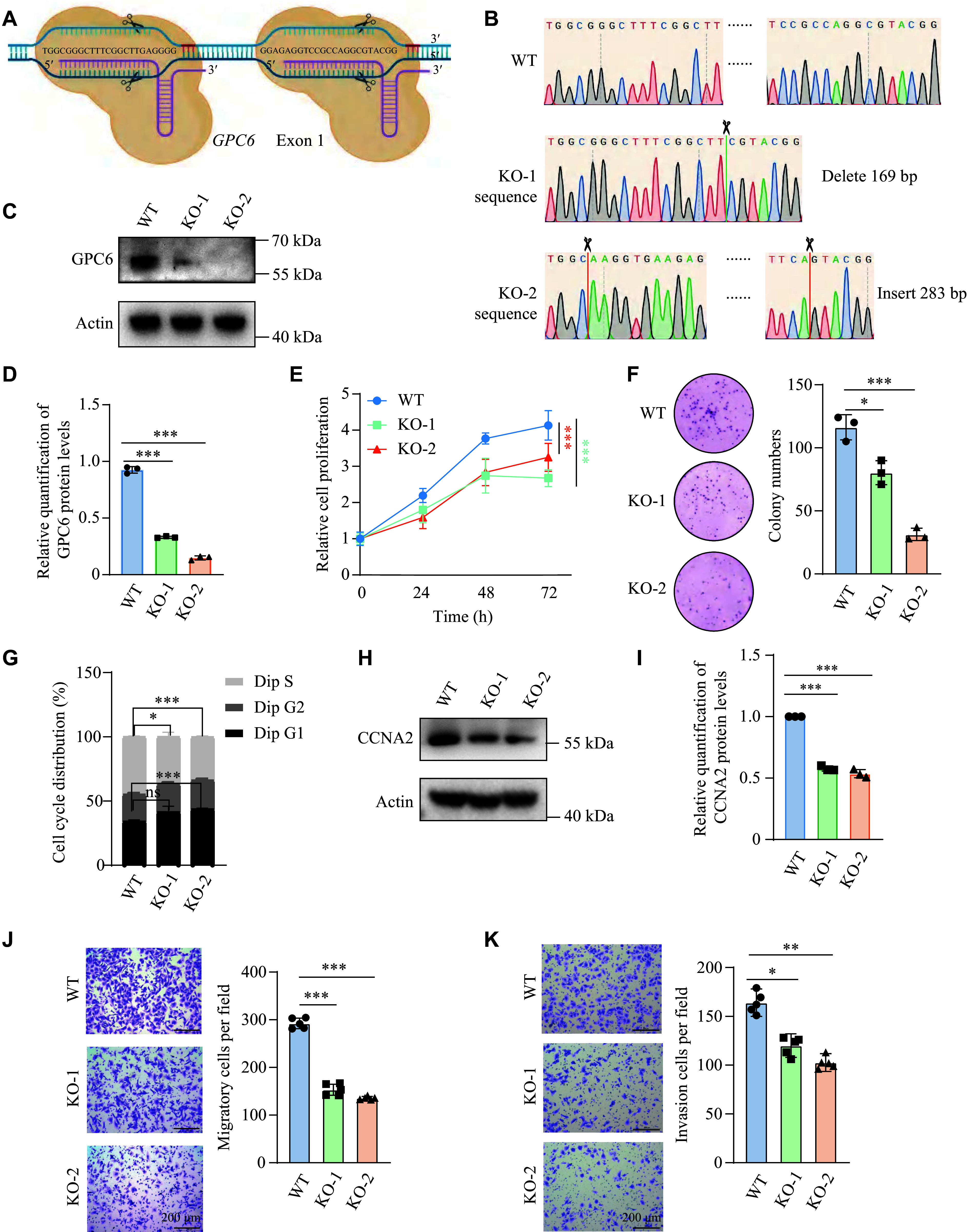
Knockout of *GPC6* hindered DAOY cell proliferation and migration. A and B: Schematic diagram (A) and sequencing results (B) of *GPC6*^−/−^ DAOY cells. C and D: Western blotting (WB) detection and quantification of GPC6 protein in wild-type (WT) and two *GPC6*^−/−^ (KO-1 and KO-2) DAOY clones. Data are shown as mean ± standard error of the mean (SEM) from three independent samples. E and F: CCK-8 assay (E) and colony formation assay (F) of WT and *GPC6*^−/−^ DAOYs; quantification data are shown as mean ± SEM from three independent experiments. G: Flow cytometry analysis of the cell cycle in WT and *GPC6*^−/−^ DAOY cells. Percentages of diploid (Dip) cells in G1, S, and G2 phases were quantified from three independent experiments. H and I: WB detection of CCNA2 protein levels of WT and *GPC6*^−/−^ DAOYs, with corresponding quantification of protein levels shown as mean ± SEM from three independent samples. J and K: Transwell migration (J) and invasion assay (K) of *GPC6*^−/−^ DAOYs; quantification data are shown as mean ± SEM from three independent experiments. ^*^P < 0.05, ^**^P < 0.01, and ^***^P < 0.001; ns represents not significant by one-way ANOVA followed by Dunnett's test compared with the WT group (D, F, G, and I–K) and two-way ANOVA followed by Dunnett's test compared with the WT group (E).

To validate our hypothesis, we generated *GPC6* knockout (*GPC6*^−/−^) DAOY cell lines using the CRISPR-Cas9 system and isolated *GPC6*^−/−^ single-cell clones (***Fig. 3A***). The knockout of *GPC6* was verified by Sanger sequencing and WB. Sanger sequencing of two single-cell clones demonstrated the presence of a 169-bp deletion or a 283-bp insertion between DNA editing sites, resulting in a frameshift mutation within the *GPC6* gene (***Fig. 3B***). WB analysis further confirmed that the single-cell clones barely expressed the GPC6 protein (***Fig. 3C*** and ***3D***). The two resulting clonal knockout cell lines were used for subsequent experiments. As expected, the proliferation ability of *GPC6*^−/−^ DAOY cells was markedly reduced compared with wild-type cells, as shown by the CCK-8 assay (***Fig. 3E***) and colony formation assay (***Fig. 3F***). The flow cytometric proliferation assay confirmed that GPC6 depletion reduced the proportion of cells in the S phase (***Fig. 3G***). In addition, the expression of cyclin A2 (CCNA2), a key activator of CDK1 and CDK2 kinases during the cell cycle, was examined. WB analysis revealed a marked reduction in CCNA2 levels in *GPC6*^−/−^ DAOY cells (***Fig. 3H*** and ***3I***). Consistently, *GPC6* knockdown using siRNAs arrested DAOY cells in the G1 stage (***Supplementary Fig. 1G***). As previously shown, *GPC6* depletion also reduced the proliferative capacity of DAOY and ONS-76 cells, whereas *GPC6* siRNAs exerted only a limited inhibitory effect on the proliferation of non-SHH-MB D283 cells (***Supplementary Fig. 1H***). Collectively, these data suggest that GPC6 promotes the growth of SHH-MB cells.

Meanwhile, the migration and invasion capabilities of DAOY cells were markedly reduced following *GPC6* knockdown (***Fig. 3J*** and ***3K***; ***Supplementary Fig. 3A*** and ***3B***). Similarly, *GPC6* knockdown in ONS-76 cells resulted in significantly impaired migration and invasion capabilities (***Supplementary Fig. 3C*** and ***3D***). Upon *GPC6* silencing, these cells exhibited elevated expression of E-cadherin, an epithelial marker, along with diminished levels of N-cadherin and MMP3, which are markers of mesenchymal cells, compared with control cells (***Supplementary Fig. 3E*** and ***3F***). Notably, *GPC6*-depleted D283 cells also displayed reduced migratory and invasive potential (***Supplementary Fig. 3G*** and ***3H***). Collectively, these results suggest that GPC6 promotes the invasiveness and metastasis of MB cells.

**Figure 4:**
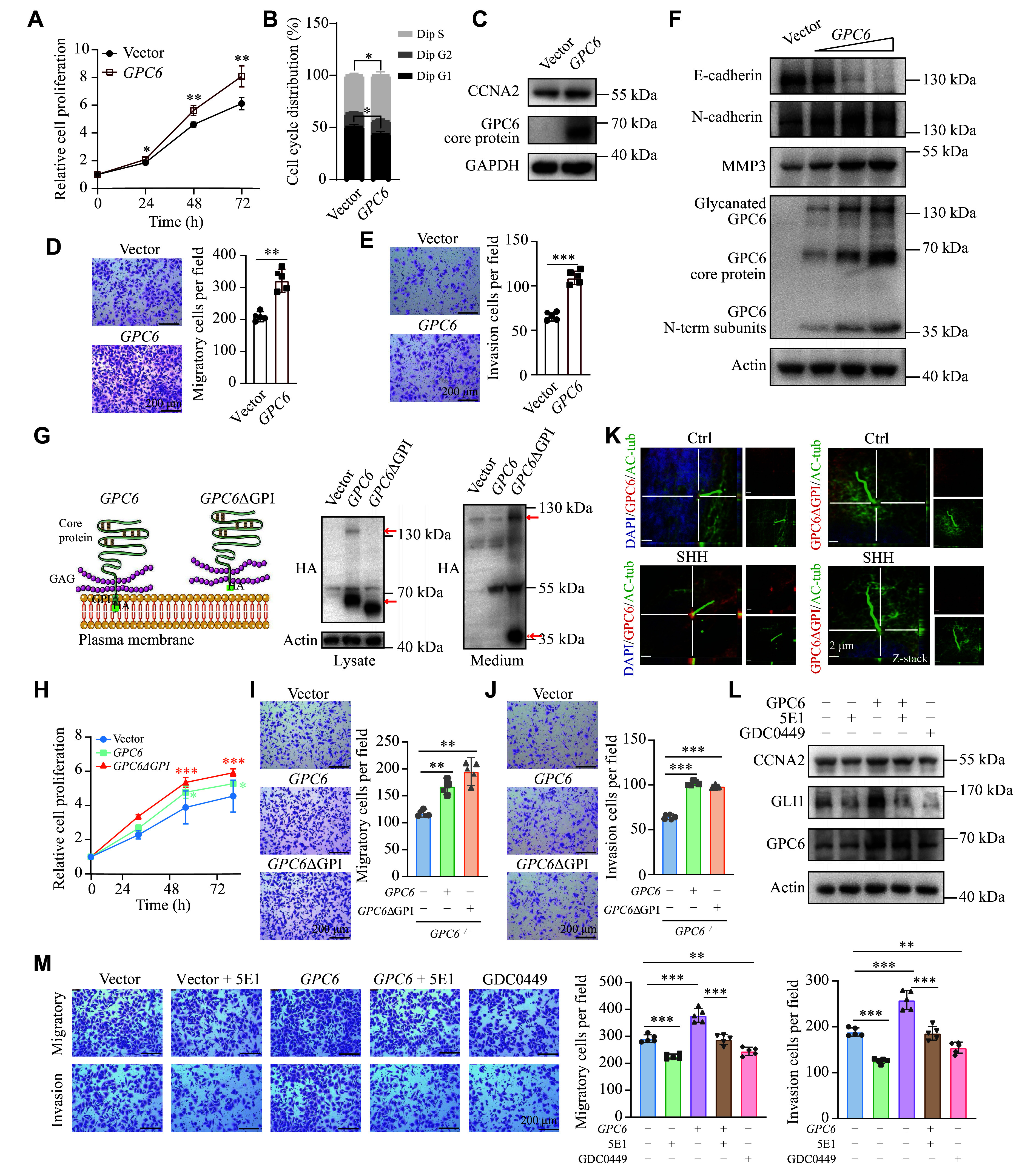
GPC6 promoted DAOY cell proliferation and migration in both anchored and secreted forms. A: Cell viability detected by CCK-8 in *GPC6*-transfected DAOYs. B: Cell cycle analysis of *GPC6*-transfected DAOYs by flow cytometry; quantification from three independent experiments. C: Western blotting (WB) detection of CCNA2 protein levels in *GPC6*-transfected DAOYs. Quantitative analyses are presented in ***Supplementary Fig. 4B***. D and E: Transwell migration (D) and invasion assays (E) of *GPC6*-transfected DAOYs; quantification data are shown as mean ± standard error of the mean (SEM) from three independent experiments. F: WB detection of GPC6 and EMT markers in DAOYs transfected with *GPC6* plasmids. Quantitative analyses are presented in ***Supplementary Fig. 4E***. G: Model of anchored and secreted GPC6 (*GPC6*ΔGPI) with WB verification. H–J: CCK-8 proliferation assay (H), transwell migration (I), and invasion assay (J) of *GPC6*^−/−^ DAOYs transfected with *GPC6* or *GPC6*ΔGPI; quantification data are shown as mean ± SEM from three independent experiments. K: Representative confocal images showing the localization of GPC6 and *GPC6*ΔGPI at the basal body of primary cilia following stimulation with exogenous SHH added to the culture medium. Ctrl, control; scale bars, 2 μm. L: WB detection of GPC6, GLI1, and CCNA2 in DAOYs transfected with *GPC6* plasmids, with SHH signaling blocked either by the SHH-neutralizing antibody 5E1 (1∶500) or the pathway inhibitor GDC0449 (100 μmol/L) for 24 h. Quantitative analyses are presented in ***Supplementary Fig. 4I***. M: Transwell migration and invasion assays of DAOYs under the same treatment conditions for 12 h. Quantification data are shown as the mean ± SEM from three independent experiments. Scale bars of panels D, E, I, J, and M, 200 μm. ^*^P < 0.05, ^**^P < 0.01, and ^***^P < 0.001 by two-way ANOVA followed by Dunnett's test compared with the vector group (A and H) and Tukey's multiple comparisons test (M), unpaired Student's *t*-test (B, D, and E), and one-way ANOVA followed by Dunnett's test compared with the vector group (I and J).

To further clarify the biological function of GPC6, we enhanced its expression in MB cells and examined its impact on cell growth, migration, and invasion. Using CCK-8 assays, we observed that increased GPC6 expression in DAOY and ONS-76 cells led to an accelerated cell growth rate (***Fig. 4A*** and ***Supplementary Fig. 4A***). Moreover, flow cytometry analysis demonstrated a notable increase in the proportion of cells in the S phase and a corresponding decrease in the G1 phase following GPC6 upregulation in DAOY cells, indicating heightened proliferation capacity (***Fig. 4B***). Consistently, elevated expression of CCNA2 was observed in *GPC6*-transfected DAOY cells (***Fig. 4C*** and ***Supplementary Fig. 4B***). Transwell assays were performed to assess the migratory and invasive potentials of DAOY and ONS-76 cells. The results revealed that cells with upregulated GPC6 displayed enhanced migration and invasion capacities (***Fig. 4D*** and ***4E***; ***Supplementary Fig. 4C*** and ***4D***). As GPC6 levels increased, these cells exhibited reduced E-cadherin expression and higher levels of N-cadherin and MMP3 (***Fig. 4F*** and ***Supplementary Fig. 4E***). The results suggest that GPC6 overexpression exacerbates the proliferation, migration, and invasion of SHH-MB cells. A similar pro-tumorigenic effect of GPC6 was also observed in D283 cells (***Supplementary Fig. 4F***–***4H***). WB analysis further detected three distinct forms of GPC6 protein within the cells (***Fig. 4F***), which are known from previous studies as N-terminal-truncated, core, and glycosylation-modified forms that progressively increase in molecular weight^[24]^.

Moreover, the introduction of exogenous *GPC6* restored the proliferation ability of *GPC6*^−/−^ DAOY cells (***Fig. 4G*** and ***4H***). Meanwhile, the defective migration and invasion capabilities of knockout cells were also rescued by the enforced expression of GPC6 (***Fig. 4I*** and ***4J***). These findings further support that GPC6 plays a role in promoting the proliferation and migration of DAOY cells. Research has indicated that GPC6 functions as a co-receptor on the cell surface, typically anchored to signal-receiving cells *via* GPI anchors^[35]^. Consistently, we observed that GPC6 localized to the base of primary cilia of DAOY cells, and its accumulation at the ciliary base was enhanced upon SHH stimulation (***Fig. 4K***; ***Supplementary Fig. 5A*** and ***5B***). SHH-MB tumors may utilize an autocrine SHH signaling mechanism, as evidenced by the pronounced inhibition of cell growth and migration upon treatment with the SMO antagonist vismodegib (GDC-0449) (***Fig. 4L*** and ***4M***; ***Supplementary Fig. 4I***)^[36]^. To determine whether GPC6-mediated cellular functions depend on SHH, we applied the SHH-neutralizing antibody 5E1. Notably, exogenous GPC6 was no longer able to promote the migration and invasion of DAOY cells following SHH blockade (***Fig. 4L*** and ***4M***; ***Supplementary Fig. 4I***), suggesting an SHH-dependent effect of GPC6 on cell migration and invasion.

To investigate whether GPI anchoring is essential for GPC6 function, we generated a mutant form, *GPC6*ΔGPI, by removing the GPI signal peptide sequence (as shown in ***Fig. 4G***) and introduced it into *GPC6*^−/−^ cells. Immunostaining results revealed that *GPC6*ΔGPI failed to localize to the ciliary base but localized around the ciliary base, unlike its wild-type counterpart (***Fig. 4K***). Notably, despite the absence of membrane anchorage, *GPC6*ΔGPI significantly rescued all the defective phenotypes of *GPC6*^−/−^ cells (***Fig. 4H–4J***), similar to wild-type GPC6. These findings suggest that GPC6 may exert its function at least partly through mechanisms independent of membrane tethering.

### GPC6 acted upstream of PTCH1 and promoted ciliogenesis

**Figure 5:**
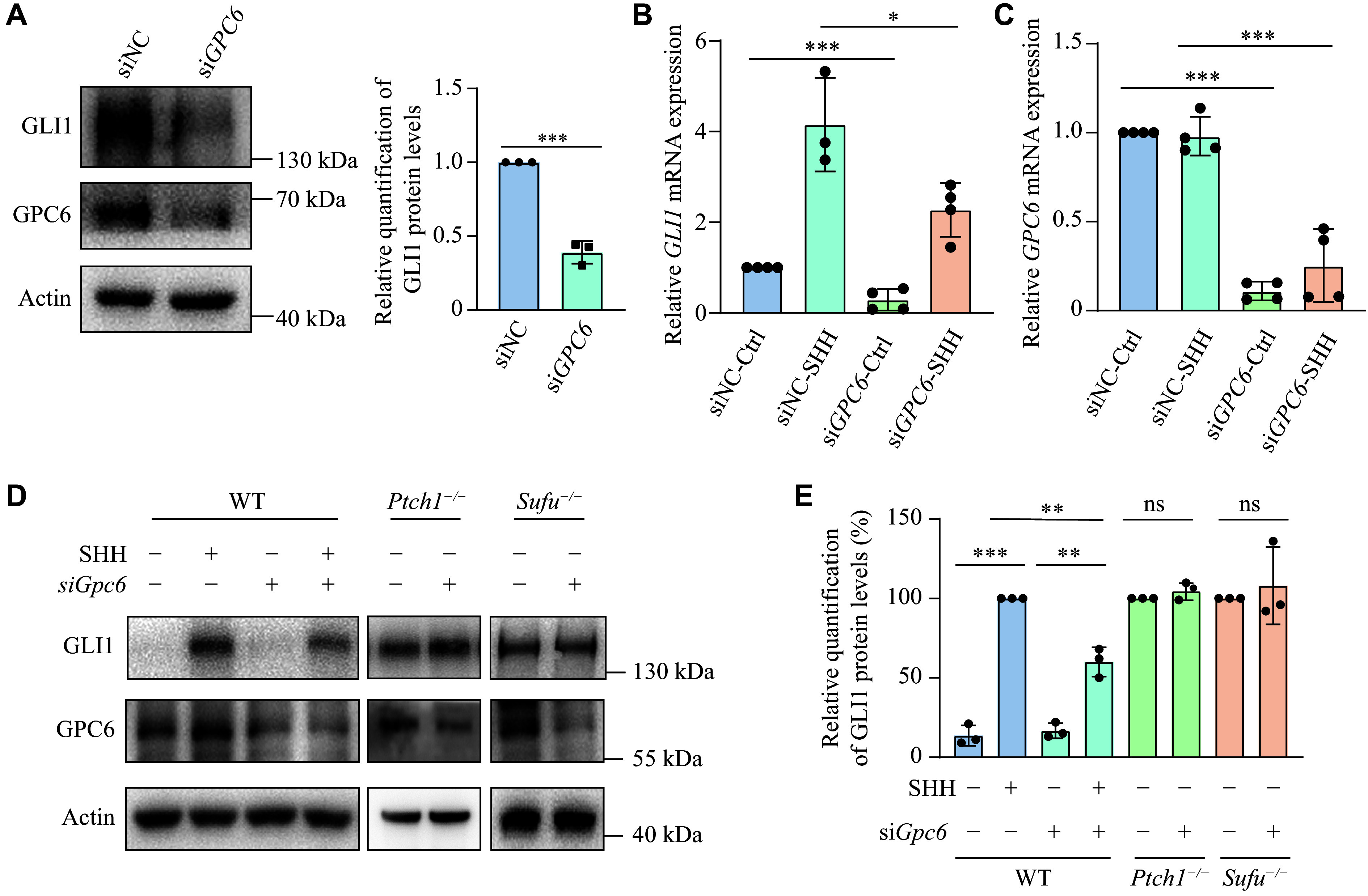
*GPC6* knockdown restricted upstream signal transduction in the SHH pathway. A: Western blotting (WB) detection of GPC6 and GLI1 in DAOYs transfected with *GPC6* siRNA (20 nmol/L) or negative control (siNC), with corresponding quantification of protein levels shown as mean ± standard error of the mean (SEM) from three independent samples. B and C: Reverse transcription-quantitative PCR analysis of *GLI1* (B) and *GPC6* (C) mRNAs in *GPC6*-knockdown DAOYs cultured in SHH-conditioned (SHH) or control medium for 24 h. D: WB analysis of GLI1 expression in WT, *Ptch1*^*−/−*^, and *Sufu*^*−/−*^ mouse embryonic fibroblasts (MEFs) transfected with *Gpc6* siRNA. Wild-type (WT) MEFs were treated with 1∶10 diluted homemade SHH-conditioned medium (SHH) for 24 h to induce GLI1 expression after siRNA transfection. E: Relative quantification of GLI1 in MEFs is shown as mean ± SEM from three independent experiments. ^*^P < 0.05, ^**^P < 0.01, and ^***^P < 0.001; ns represents not significant by unpaired Student's *t*-test (A and E for *Ptch1^−/−^* and *Sufu^−/−^* MEFs), and two-way ANOVA followed by Tukey's multiple comparisons test (B, C, and E for WT MEFs).

It is known that SHH-MBs are characterized by the pathogenic activation of SHH signaling. GPC6 has been reported to stimulate Indian Hedgehog (IHH) signaling by interacting with IHH through its core protein and with PTCH1 through its glycosaminoglycan chains in the developing long bones of embryos^[22]^. To investigate whether GPC6 plays a comparable role in the signal transduction of DAOY cells, we first assessed SHH signaling activity in *GPC6*-depleted cells. The notable reduction in GLI1 protein levels indicated disrupted SHH signaling in the absence of *GPC6* (***Fig. 5A***). Remarkably, DAOY cells responded to SHH stimulation, but this response was markedly impaired in the absence of GPC6 (***Fig. 5B*** and ***5C***). Consistent with these findings, WB analyses of MEFs revealed that reduced levels of GPC6 hindered SHH signal reception (***Fig. 5D*** and ***5E***). This suggests that GPC6 plays a facilitating role in SHH signal transduction. To more precisely determine where GPC6 acts in the SHH pathway, *Gpc6* was knocked down with siRNA in mutant MEF cells lacking different SHH signaling repressors. The SHH pathway is continuously activated in *Ptch1*^−/−^ and *Sufu*^−/−^ MEFs in the absence of the SHH ligand, and siRNA depletion of *Gpc6* failed to reduce GLI1 levels in *Ptch1*^−/−^ and *Sufu*^−/−^ MEFs, suggesting that GPC6 acts upstream in SHH signaling (***Fig. 5D*** and ***5E***).

**Figure 6:**
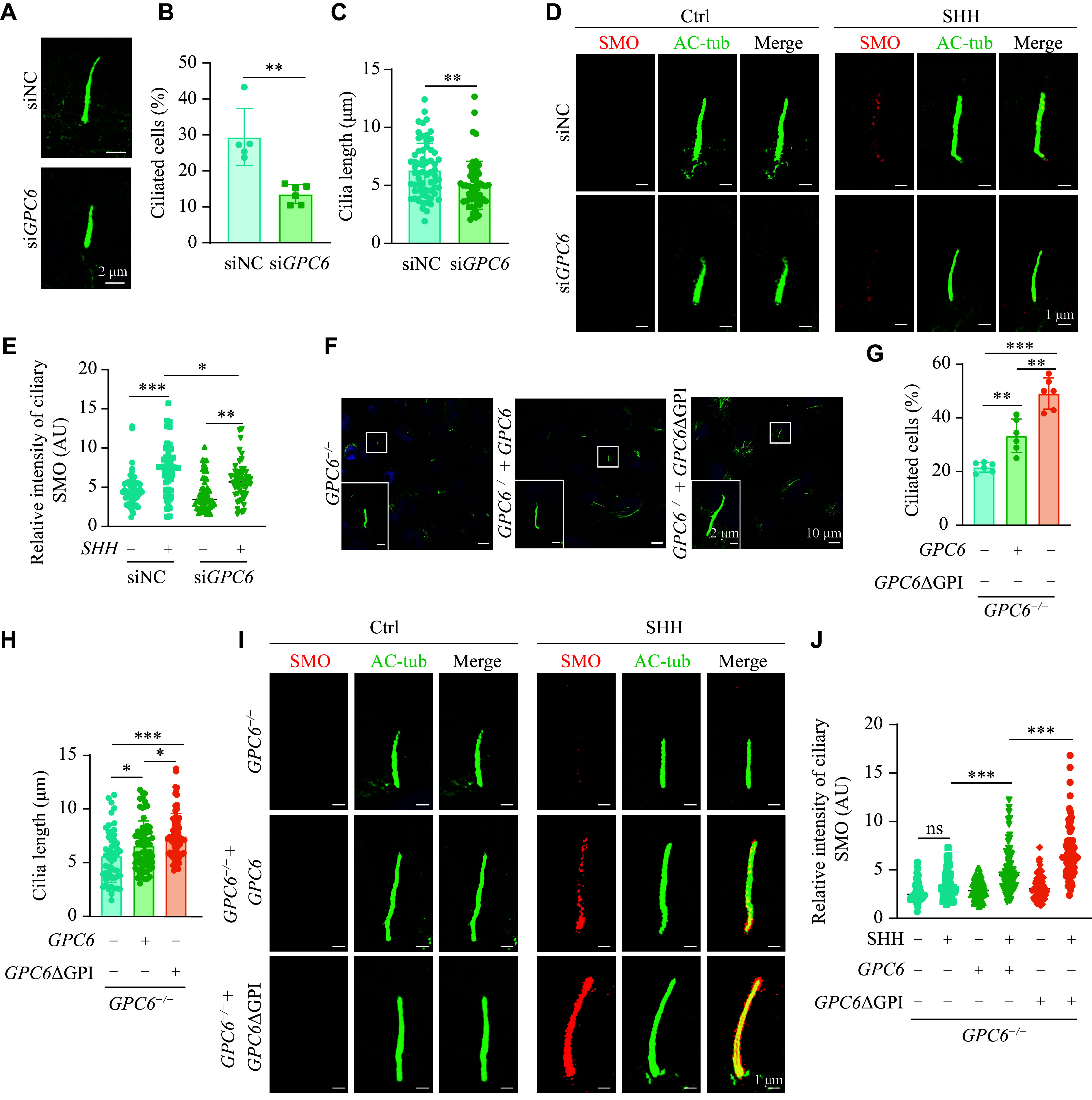
GPC6 localized at the basal body of the primary cilium and promoted its formation and growth. A: Representative confocal images of anti-acetylated tubulin (AC-tub; green) showing the primary cilium in DAOYs transfected with *GPC6* siRNA (20 nmol/L), with siNC as the control. Cells were starved in 0.5% FBS/DMEM for 24 h for ciliogenesis. Nuclei stained with DAPI (blue). Scale bars, 2 μm. B and C: Quantitation of percentage (B) (*n* ≥ 5 in each group) and ciliary length (C) (*n* > 50 in each group) of ciliated cells presented in (A). D: Representative immunofluorescence staining of SMO protein in primary cilia of DAOYs transfected with *GPC6* siRNA, with siNC as the control, and treated with 1∶10 diluted homemade SHH conditioned medium (SHH) or not (Ctrl) in 0.5% FBS/DMEM for 24 h. Scale bars, 1 μm. E: Quantification of SMO fluorescence intensity in cilia in (D) (*n* ≥ 50 in each group). F: Representative confocal images of anti-acetylated tubulin (green) showing primary cilium in *GPC6*^−/−^ DAOYs infected with *GPC6* or *GPC6*ΔGPI lentivirus, with *GPC6*^−/−^ as the control. Scale bars, 2 μm (insets) and 10 μm (main images). G and H: Quantitation of percentage (G) (*n* ≥ 5 in each group) and ciliary length (H) (*n* > 50 in each group) of ciliated cells presented in (F). I: Immunofluorescence staining of SMO protein in primary cilia of *GPC6*^−/−^ DAOYs infected with *GPC6* or *GPC6*ΔGPI lentivirus and treated with SHH conditioned medium, with *GPC6*^−/−^ as the control. Scale bars, 1 μm. J: Quantification of SMO fluorescence intensity in cilia in (I) (*n* ≥ 50 in each group). Data in bar graphs are expressed as mean ± standard error of the mean (SEM). ^*^P < 0.05, ^**^P < 0.01, and ^***^P < 0.001; ns represents not significant by unpaired Student's *t*-test (B and C), two-way ANOVA followed by Tukey's multiple comparisons test (E and J), or one-way ANOVA followed by Tukey's multiple comparisons test (G and H).

Since GPI anchoring is not essential for GPC6 function, we next explored the potential roles of GPC6 beyond its co-receptor function. Our initial focus was to investigate ciliogenesis, as the primary cilium is a crucial subcellular site where the initial steps in SHH signal transduction occur in vertebrate cells^[37]^. Knocking down *GPC6* in DAOY cells led to a significant reduction in the proportion of ciliated cells (***Fig. 6A*** and ***6B***). Moreover, the cilia in *GPC6* knockdown cells were shorter than those in control cells (***Fig. 6A*** and ***6C***). Immunofluorescent results also indicated that the SHH-induced accumulation of endogenous SMO in the primary cilia was substantially impaired in *GPC6* knockdown DAOY cells (***Fig. 6D*** and ***6E***). Furthermore, in *GPC6*-deficient DAOY cells, restoring the expression of GPC6 or its mutants rescued the ciliary defects (***Fig. 6F–6H***), with a concomitant restoration of SHH-induced accumulation of endogenous SMO in the primary cilia (***Fig. 6I*** and ***6J***). These data confirm that compromised cilia formation results from the absence of GPC6, suggesting that GPC6 promotes ciliogenesis independently of its anchorage to the cell membrane.

### GPC6 participated in the secretion of SHH through extracellular vesicles

**Figure 7:**
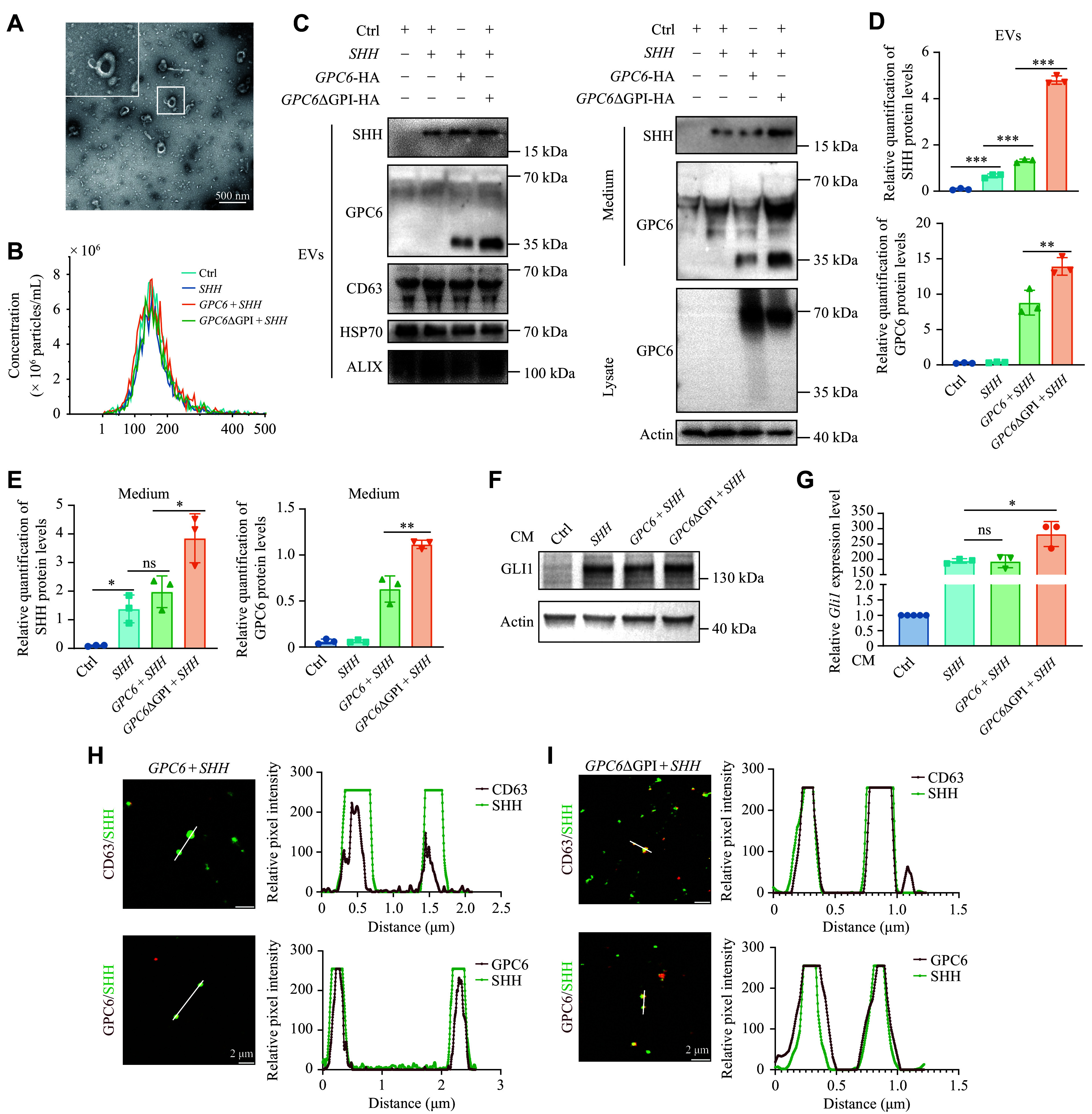
GPC6ΔGPI enhanced the secretion of SHH through extracellular vesicles (EVs). A: Representative electron microscopic image of EVs derived from 293T cells. Scale bars, 500 nm. B: Average size distribution of EVs measured by nanoparticle tracking analysis (NTA). C–E: Western blotting analysis (C) of SHH and GPC6 in EVs, conditioned medium, and cell lysates from 293T cells transfected with empty vector (Ctrl), *GPC6*, *GPC6*ΔGPI, and *SHH*-N plasmids. CD63, heat shock protein 70 (HSP70), and ALIX serve as EV markers, with corresponding quantification of protein levels (D and E) shown as mean ± standard error of the mean (SEM) from three independent samples. F and G: Expression levels of GLI1 protein (F) and mRNA (G) in mouse embryonic fibroblasts (MEFs) cultured with conditioned medium (CM) from transfected 293T cells (C). Data in bar graphs are presented as mean ± SEM. H and I: Immunostaining of EVs from GPC6- (H) or *GPC6*ΔGPI- (I) expressing 293T cells showing SHH (green), CD63 (red), and GPC6 (red). Scale bars, 2 μm. Line scan determination of the white bars. ^*^P < 0.05, ^**^P < 0.01, and ^***^P < 0.001; ns represents not significant by one-way ANOVA followed by Tukey's multiple comparisons test (D, E, and G).

Some GPC family members are reported to be secreted without GPI anchoring when anchors are limited^[38]^. This suggests that the secreted form of GPC6 may promote SHH-MB development by facilitating the secretion of the SHH ligand. To investigate this, *GPC6* and *GPC6*ΔGPI constructs were co-transfected with *SHH*-N plasmids into 293T cells, followed by the isolation of EVs from the conditioned medium using differential ultracentrifugation. Electron microscopy revealed that the EVs maintained intact membrane-enclosed structures (***Fig. 7A***). Nanoparticle tracking analysis showed a prominent peak with an average diameter of 180 nm (***Fig. 7B***). WB analysis further confirmed the presence of the exosomal marker CD63 in these EVs (***Fig. 7C***). Notably, the presence of GPC6ΔGPI led to increased detection of SHH-N within the EVs without altering their dimensions, suggesting that GPC6ΔGPI enhances SHH-N secretion (***Fig. 7C***–***7E***). MEFs, which are sensitive to SHH signaling, were treated with the conditioned medium for 24 h and exhibited a more pronounced upregulation of GLI1 expression (***Fig. 7F*** and ***7G***), indicating that the EVs containing GPC6ΔGPI carried more SHH ligand. It was also observed that GPC6ΔGPI appeared in a truncated form in EVs (***Fig. 7C***). Furthermore, immunostaining of EVs showed the colocalization of SHH-N with GPC6 and CD63, as supported by line scan analysis (***Fig. 7H*** and ***7I***). These findings collectively suggest that the non-anchored form of GPC6 has a greater capacity to promote SHH secretion.

**Figure 8:**
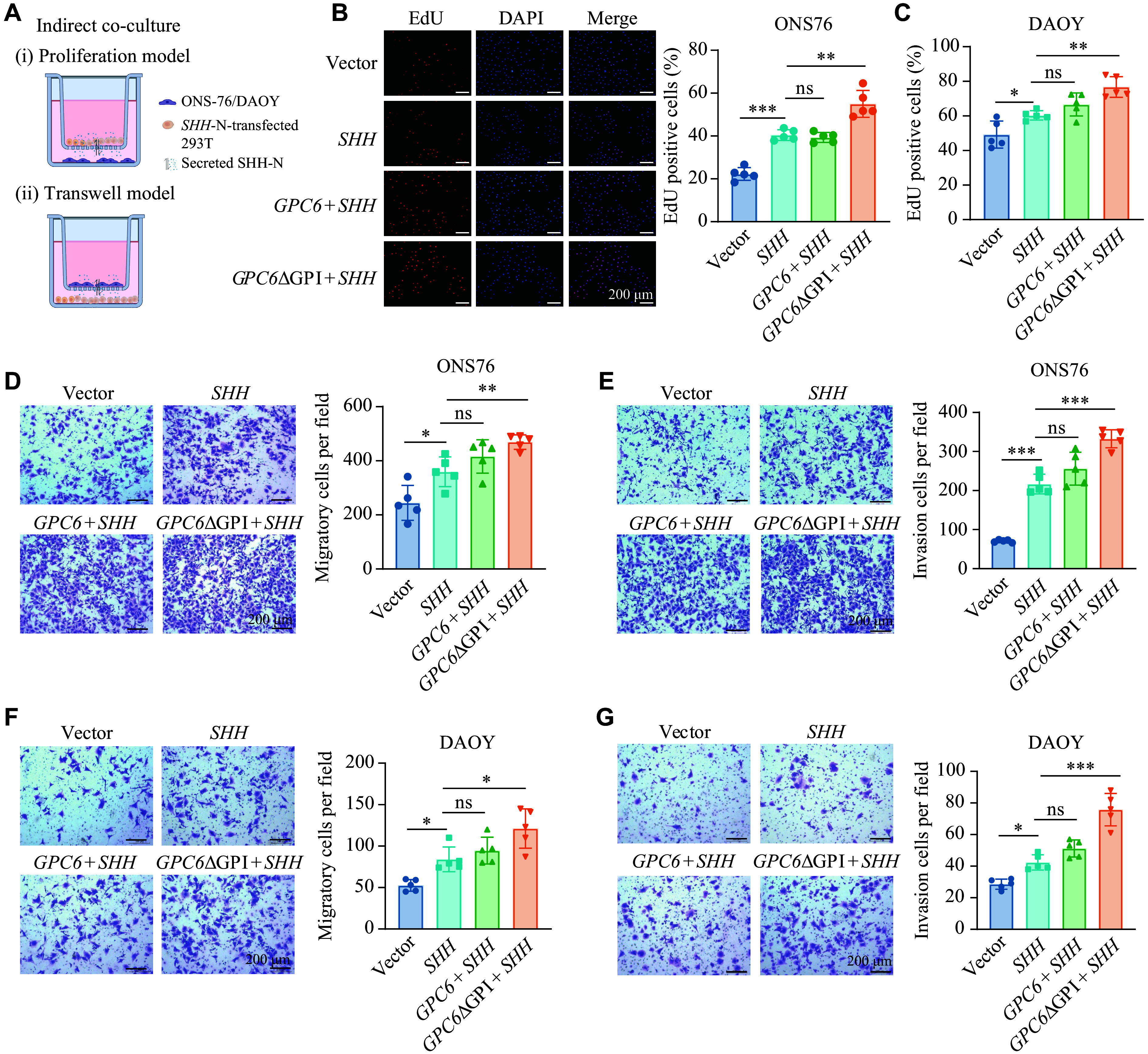
GPC6ΔGPI promoted SHH-induced proliferative and migratory potential of ONS-76 cells. A: Schematic representation of the indirect co-culture system: (i) Proliferation model: ONS-76 or DAOY cells were plated in the lower chamber, and 293T cells transfected with Vector, SHH-N, GPC6, and GPC6ΔGPI plasmids were plated in the upper chamber. (ii) Transwell model: ONS-76 or DAOY cells were plated in the upper chamber (coated with Matrigel for invasion), and transfected 293T cells were plated in the lower chamber. B: Representative EdU staining images of ONS-76 cells in the lower chamber (i) and quantification of the proliferating cells in the systems. C: Quantification of the proliferating DAOY cells in the co-culture system by EdU assay. D and E: Transwell migration (D) and invasion assay (E) of ONS-76 cells in the upper chamber (ii), with quantification of the migratory and invading cells in the systems. F and G: Transwell migration (F) and invasion assay (G) of DAOY cells in the upper chamber (ii), with corresponding quantification. Scale bar, 200 µm. Data in bar graphs are shown as mean ± standard error of the mean (SEM) (*n* = 3). ^*^P < 0.05, ^**^P < 0.01, and ^***^P < 0.001; ns represents not significant by two-way ANOVA followed by Tukey's multiple comparisons test (B–G).

To evaluate the effects of communication between SHH-producing cells and MB cells *in vitro*, an indirect co-culture system was established (***Fig. 8A***). As expected, the conditioned medium from 293T cells expressing *SHH*-N with *GPC6*ΔGPI promoted the proliferation of ONS-76 and DAOY cells more effectively than *SHH*-N alone, as demonstrated by EdU incorporation results (***Fig. 8B*** and ***8C***). Transwell assays further confirmed that *SHH*-N with *GPC6*ΔGPI significantly enhanced the migration and invasion of ONS-76 (***Fig. 8D*** and ***8E***) and DAOY cells (***Fig. 8F*** and ***8G***). The findings suggest that the truncated form of GPC6 promotes the release of more SHH-N in extracellular vesicles, leading to enhanced proliferative and migratory capacities of ONS-76 and DAOY cells in response to SHH-N signaling.

## Discussion

GPCs are evolutionarily conserved across diverse animal species and bind to various soluble and insoluble ligands, playing crucial roles in regulating multiple cellular functions, including morphogenesis and tissue homeostasis^[17]^. Dlp is required for Hedgehog signal reception, whereas Dally is essential for the long-range transport of Hedgehog in *Drosophila*^[16]^. It has also been shown that GPCs exhibit both stimulatory and inhibitory effects on mammalian SHH signaling. For instance, GPC3 interacts with SHH independently of PTCH1 at the cellular membrane, sequestering the ligand and attenuating signaling^[39]^. A recent study demonstrated that GPC3-associated heparan sulfate plays an essential role in modulating Hedgehog signaling, specifically at a downstream step involving the GLI transcription factors rather than at the ligand reception level as previously assumed^[40]^. In contrast, GPC5 at the plasma membrane interacts with both SHH and PTCH1 proteins, thereby facilitating and enhancing the SHH-PTCH1 interaction. This interaction amplifies the signaling cascade, ultimately resulting in enhanced signaling efficiency^[41]^. The *GPC6* gene is located on chromosome 13q31.3, closely clustered with the *GPC5* gene in the genome^[20]^. This proximity may reflect their functional coevolution and co-regulation. Notably, SHH-MB is characterized by aberrant activation of the SHH signaling pathway. Among the glypican family, only GPC6 was found to promote tumor cell growth in our study, while GPC3 and GPC5 were minimally expressed, and GPC1 had no significant impact on proliferation.

The growth-promoting effect of GPC6 on MB primarily depends on its ability to sustain SHH signaling, a function also observed during development. GLI1 and GPC6 exhibit synchronized expression patterns in the developing cerebellum, with a similar co-expression pattern observed in MB tissues. However, the causal relationship between these two genes remains to be elucidated. Similar findings have been reported in the development of long bones, the gastrointestinal tract, and the heart. Loss-of-function mutations of *GPC6* lead to autosomal recessive omodysplasia type 1 (OMOD1), characterized by short stature, shortened limbs, and facial dysmorphism^[22]^. *GPC6*^−/−^ mouse embryos display many of the developmental abnormalities in OMOD1 patients^[22]^. Smaller stomachs and shorter small intestines are also observed in *GPC6*^−/−^ embryos^[23–24]^. More notably, *GPC6*^−/−^ mouse embryos exhibit severe cardiac malformations, namely a tetralogy of Fallot (TOF)-like defect, including double outlet right ventricle (DORV) with a rightward malpositioned aorta, perimembranous ventricular septal defect (VSD), right ventricular (RV) hypertrophy, and narrowing of the pulmonary artery^[42]^. These phenotypes observed in *GPC6*^−/−^ mice highlight GPC6 as an important extracellular matrix component in developmental signaling pathways, such as Hedgehog and WNT.

To date, there has been limited evidence indicating the involvement of glypicans in the secretion and transport of SHH. The current study demonstrates that GPC6 enhances SHH signaling through multiple mechanisms: acting as a co-receptor to promote signal transduction, supporting ciliogenesis (which is critical for SHH reception), and facilitating SHH ligand secretion *via* extracellular vesicles. The truncated, secreted form of GPC6 further amplifies SHH signaling by increasing SHH ligand availability. These findings underscore GPC6 as a key driver of SHH-MB cell proliferation, migration, and invasion by modulating both intracellular signaling and the tumor microenvironment.

Beyond SHH-MB, GPC6 has been implicated in other malignancies, including gastric cancer, cutaneous melanoma, and nasopharyngeal carcinoma, though its precise mechanisms remain unclear^[43–45]^. Interestingly, studies in cardiac development further support GPC6's role in Hedgehog signaling regulation^[46]^. Hedgehog signaling is essential for early heart morphogenesis, as demonstrated by Hedgehog knockout mice, which exhibit embryonic lethality and characteristic TOF-like cardiac defects, including ventricular septal defects and overriding aorta^[47–48]^. In the developing heart, GPC6 is expressed in the endocardial cushion, and its deficiency leads to defects that resemble those caused by impaired Hedgehog signaling^[42]^. Additionally, recent studies highlight the interaction between GPC6 and ADAMTS1/5 metalloproteases^[49]^. Combined inactivation of these two matrix-degrading metalloproteases leads to TOF-like abnormalities. Mechanistic investigations revealed that ADAMTS1 and ADAMTS5 mediate specific cleavage events in GPC6, as a cleaved GPC6 peptide was detected exclusively in control hearts but not in ADAMTS1/5 knockout hearts^[49]^. Paradoxically, the loss of both *ADAMTS* enzymes resulted in reduced cardiac GPC6 levels and decreased *Gpc6* transcription in the outflow tract. This reduction disrupts Hedgehog signaling, thereby contributing to outflow tract malrotation and associated structural defects^[49]^. These findings suggest that, in addition to the full-length form of GPC6 on the cell surface, the N-terminal truncated form of GPC6 in the extracellular matrix also plays a role in Hedgehog signal transduction in both cardiac development and disease.

**Figure 9:**
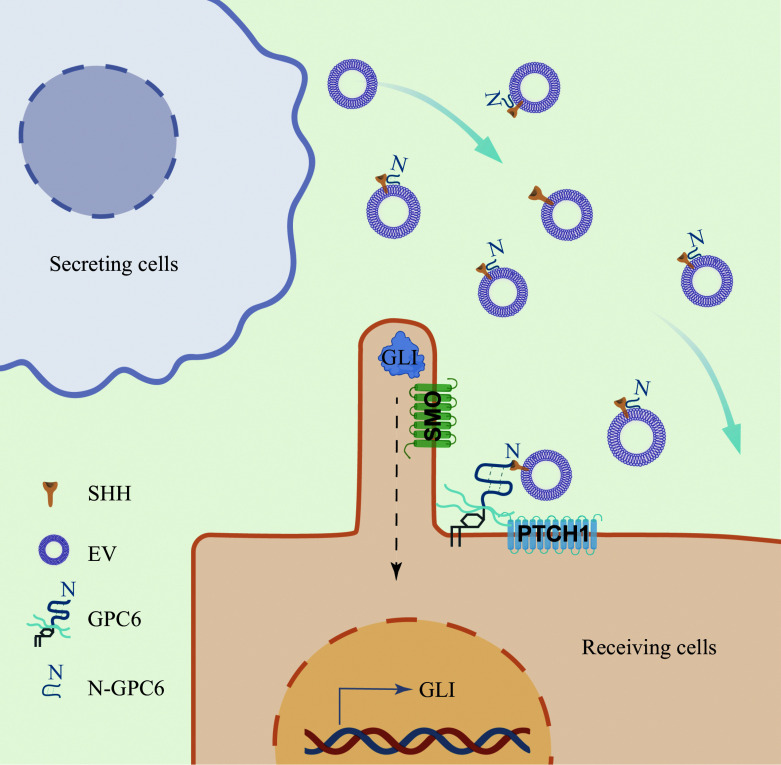
Proposed mechanism of glypican-6 (GPC6)-driven Hedgehog ligand secretion and signaling response in medulloblastoma. GPC6 participates in the interaction between Sonic Hedgehog (SHH) and Patched 1 (PTCH1) in receiving cells to promote SHH signaling. In addition, in secreting cells, N-terminal GPC6 promotes SHH secretion through extracellular vesicles (EVs).

In conclusion, GPC6 functions as a pivotal regulator of SHH signaling, contributing to both normal developmental processes and pathological conditions. Its dual function, acting as a co-receptor at the cell surface and facilitating SHH secretion *via* extracellular vesicles, underscores its versatility in modulating the Hedgehog pathway (***Fig. 9***). The discovery of GPC6's role in SHH-MB provides new insights into its biological function and highlights it as a potential therapeutic target for glypican-mediated oncogenic signaling.

## Additional information

The online version contains supplementary materials available at http://www.jbr-pub.org.cn/article/doi/10.7555/JBR.39.20250406?pageType=en.
