# Supplementary material for: GPC6 facilitates progression of SHH-subgroup medulloblastoma by enhancing Hedgehog secretion and signaling responses
Source: J Biomed Res. 2026 May 21;40(3):228–46. doi: 10.7555/JBR.39.20250406 (PMC13231367; doi:10.7555/JBR.39.20250406)
Supplement: Supplementary file 1 — The online version contains supplementary materials available at http://www.jbr-pub.org.cn/article/doi/10.7555/JBR.39.20250406?pageType=en. [file jbr-40-3-228-S1.pdf]

Available online at www.jbr-pub.org.cn

Open Access at PubMed Central

Journal of Biomedical Research, 2026 40(3): 1–7

Supplementary Data

JBR

GPC6 facilitates progression of SHH-subgroup medulloblastoma by enhancing Hedgehog secretion and signaling responses

Yue Wang[1,2,△], Qingyue Meng[1,△], Qin Zhu[1], Xinyi Zhang[1], Xinfa Wang[3], Junping He[3], Jing Cai[1], Xiaohong Pu[4], Zihe Ai[1], Qinya Li[1], Kedui Pu[1], Tingting Yu[1,✉], Chen Liu[1,✉], Shen Yue[1,✉]

[1]*Department of Medical Genetics, Nanjing Medical University, Nanjing, Jiangsu 211166, China;*
[2]*Institute of Geriatric Medicine, Jiangsu Province Geriatric Hospital, Nanjing, Jiangsu 210009, China;*
[3]*Department of Neurosurgery, Children's Hospital of Nanjing Medical University, Nanjing, Jiangsu 210093, China;*
[4]*Department of Pathology, Nanjing Drum Tower Hospital, the Affiliated Hospital of Nanjing University Medical School, Nanjing, Jiangsu 210008, China.*

Supplementary Table 1 **Targeting sequences for sgRNAs**

Names	Sequences (5′–3′)
Site A-sgRNA	CACCGTGGCGGGCTTTCGGCTTGAG
Site A-sgRNA-rc	AAACCTCAAGCCGAAAGCCCGCCAC
Site B-sgRNA	CACCGGAGAGGTCCGCCAGGCGTA
Site B-sgRNA-rc	AAACTACGCCTGGCGGACCTCTCC

△These authors contributed equally to this work.

✉Corresponding authors: Shen Yue, E-mail: yueshen@njmu.edu.cn, ORCID: 0000-0002-3194-1757; Chen Liu, liuchen@njmu.edu.cn, ORCID: 0000-0002-7134-4367; Tingting Yu, tingting@njmu.edu.cn, ORCID: 0000-0003-1694-7592.

Received: 23 September 2025; Revised: 23 November 2025; Accepted: 23 November 2025; Available online: 28 November 2025; Published date: 21 May 2026

CLC number: R739.41, Document code: A

The authors reported no conflict of interests.

This is an open access article under the Creative Commons Attribution (CC BY 4.0) license, which permits others to distribute, remix, adapt and build upon this work, for commercial use, provided the original work is properly cited.

© 2026 by Journal of Biomedical Research.

https://doi.org/10.7555/JBR.39.20250406

Supplementary Table 2 **Primers for reverse transcription-quantitative PCR**

Genes	Sequences (5′–3′)
Homo-*GPC6*-F	GAGAAGTTGCAAACCGAGTTTC
Homo-*GPC6*-R	ATGACGTTGAGACAGTAGTTGT
Homo-*GPC5*-F	GCTGTATTTATTTGGTGCGGAT
Homo-*GPC5*-R	AGGGTTAATGAGGTGGTTGTAG
Homo-*GPC4*-F	AAGAATGCATACAATGGGAACG
Homo-*GPC4*-R	GTGGCATTGTAGTCAAACTCTG
Homo-*GPC3*-F	TTGCAAGTATGTCTCCCTAAGG
Homo-*GPC3*-R	TGTTCAATCGTGCTGTTAGTTG
Homo-*GPC2*-F	GCTTCTGCTGCTGCCTCTGTG
Homo-*GPC2*-R	AGGGCGGGAGGGATTAGGTTTAAG
Homo-*GPC1*-F	CTATTGCCGAAATGTGCTCAAG
Homo-*GPC1*-R	GATGTACCCCAGAACTTGTCG
Homo-*GLI1*-F	AACCCTTGGAAGGTGATATGTC
Homo-*GLI1*-R	TTCATACACAGATTCAGGCTCA
Homo-*β-ACTIN*-F	CATCGAGCACGGCATCGTCA
Homo-*β-ACTIN*-R	TAGCACAGCCTGGATAGCAAC
Mus-*Gli1*-F	GGTGTGTAATTACGTTCAGTCG
Mus-*Gli1*-R	GGATAGGAGCCTGATTTGTGAT
Mus-*β-Actin*-F	CATTGCTGACAGGATGCAGAAGG
Mus-*β-Actin*-R	TGCTGGAAGGTGGACAGTGAGG

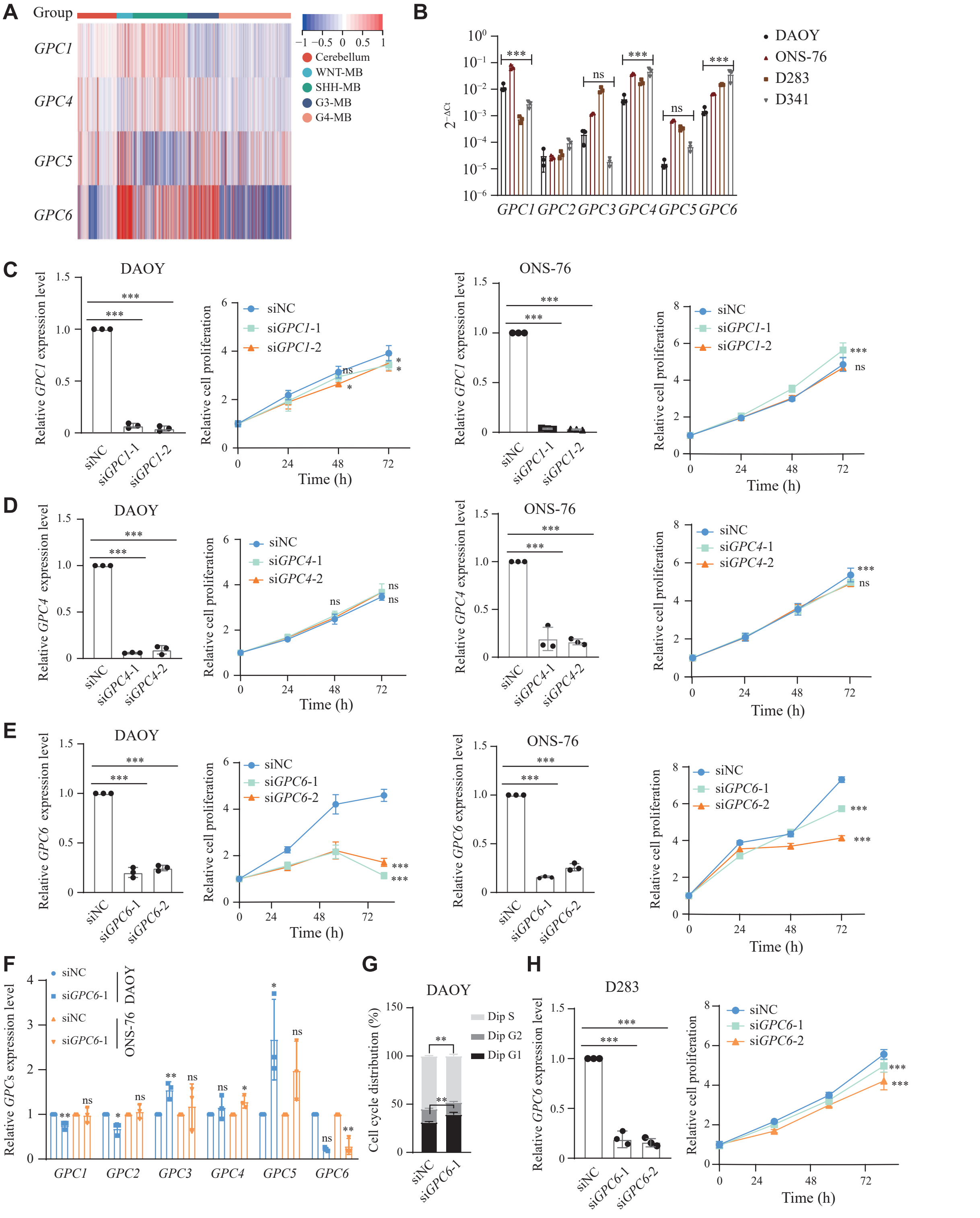

Supplementary Fig. 1 **Knockdown of *GPC6* hindered the growth of medulloblastoma (MB) cells.** A: Comparison of mRNA abundance of glypicans in normal cerebella (n = 291), WNT subgroup MB (n = 118), SHH subgroup MB (n = 405), Group 3 subgroup MB (n = 233), and Group 4 subgroup MB (n = 530). Data from the GSE124814 dataset. B: Relative mRNA expression levels of *GPC*s (*GPC1*–*GPC6*) in MB cell lines DAOY, ONS-76, D283, and D341, determined by reverse transcription-quantitative PCR (RT-qPCR). Data are presented as $2^{-\Delta Ct}$ values. Expression levels were normalized to *GAPDH*, and the lowest-expressed gene, *GPC2*, was used as the reference for comparison across family members. C–E: Cell proliferation of DAOY and ONS-76 cells transfected with *GPC1*, *GPC4*, or *GPC6* siRNAs (20 nmol/L) was evaluated using the CCK-8 assay. Knockdown efficiency was confirmed by RT-qPCR. F: RT-qPCR analysis of mRNA expression levels of all six glypicans in DAOY and ONS-76 cells following the knockdown of *GPC6*. Quantification data are shown as mean ± standard error of the mean (SEM) from three independent experiments. G: Flow cytometry analysis for cell cycle distribution of *GPC6*-knockdown DAOY cells. H: Cell proliferation of non-SHH-MB D283 cells transfected with *GPC6* siRNAs was evaluated using the CCK-8 assay. Knockdown efficiency was confirmed by RT-qPCR. Quantification data are shown as mean ± SEM from three independent experiments. $^{*}P < 0.05$, $^{**}P < 0.01$, and $^{***}P < 0.001$, and ns represents not significant, by one-way ANOVA (B), one-way ANOVA followed by Dunnett's test compared with the siNC group (RT-qPCR data from C–E and H), two-way ANOVA followed by Dunnett's test compared with the siNC group (CCK-8 data from C–E and H), and unpaired t-test (F and G).

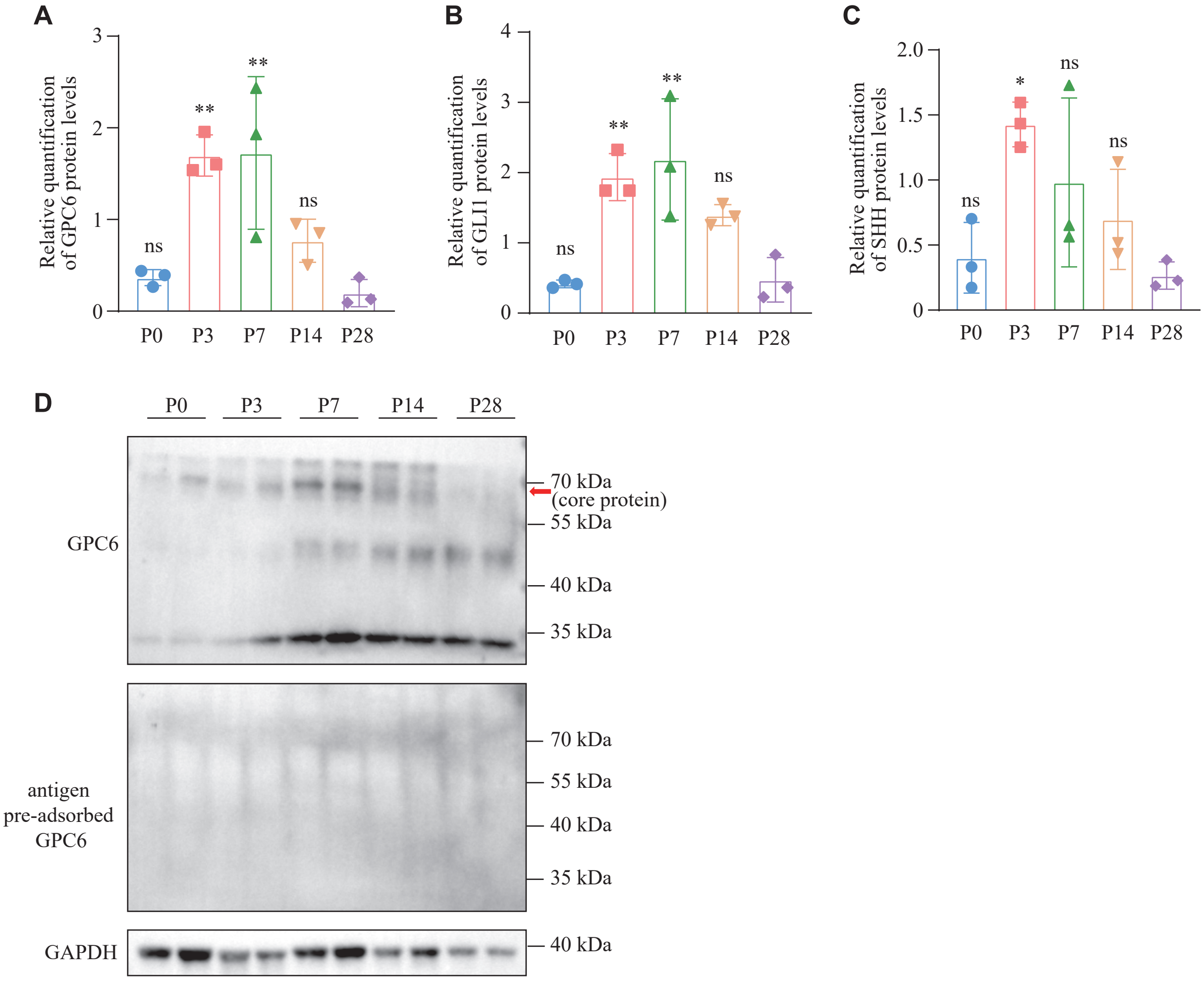

Supplementary Fig. 2 **Identification of different GPC6 isoforms in the developing mouse cerebellum.** A–C: Relative quantification of GPC6, GLI1, and SHH protein levels during cerebellar development in normal mice, as shown in ***Fig. 2C***. Data are presented as mean ± standard error of the mean (SEM) from three independent experiments. D: Western blotting detection of GPC6 protein levels during cerebellar development of wild-type mice, using an anti-GPC6 antibody (top) and the same antibody pre-adsorbed with GPC6 peptide (middle), confirming the specificity of the detected bands. $^{*}P < 0.05$, $^{**}P < 0.01$, and ns represents not significant, by one-way ANOVA followed by Dunnett's test compared with the P28 group (A–C).

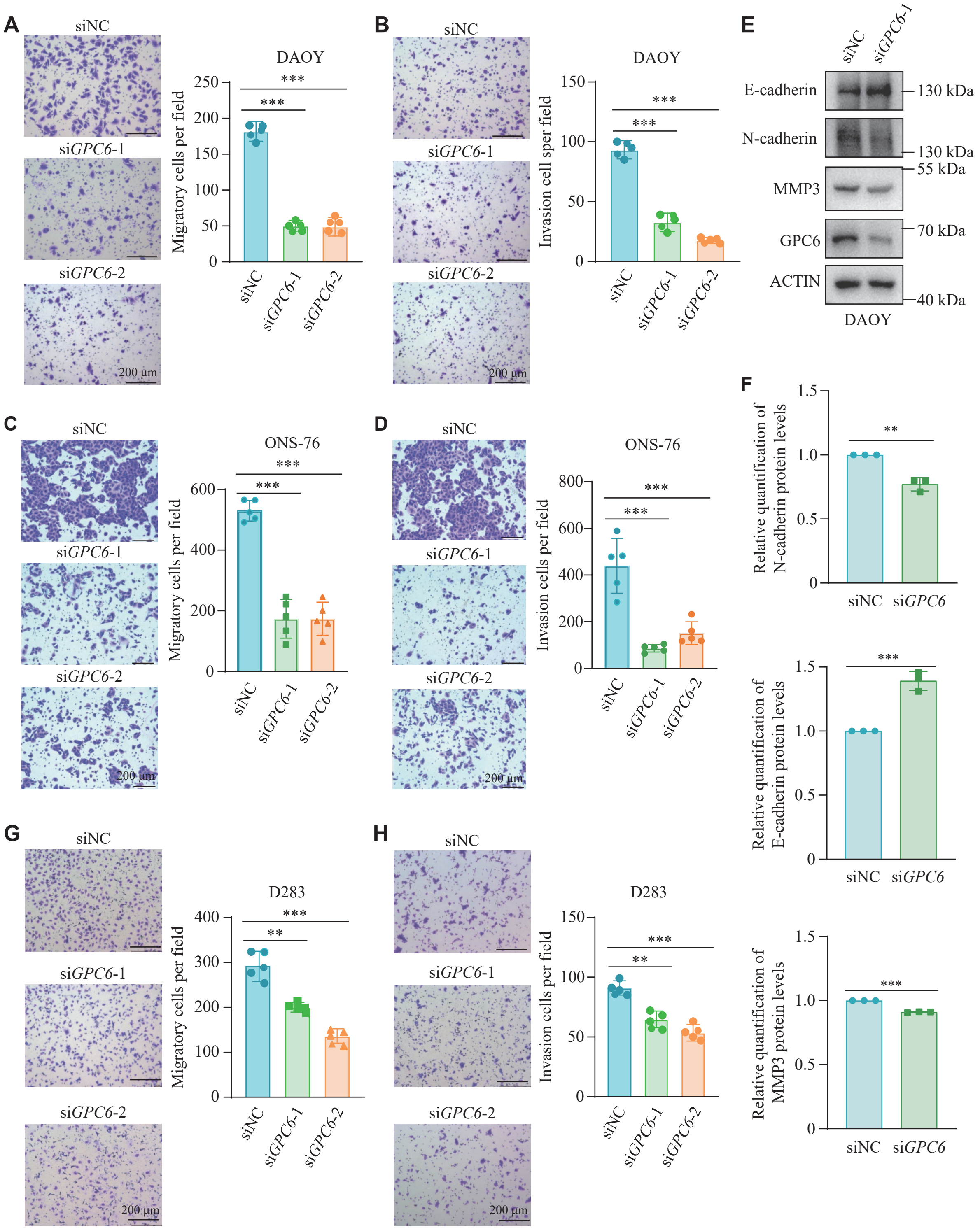

Supplementary Fig. 3 **Knockdown of *GPC6* hindered SHH-MB cell migration.** A and B: Transwell migration (A) and invasion assays (B) of *GPC6*-knockdown (si*GPC6*) DAOY cells. C and D: Transwell migration (C) and invasion assays (D) of *GPC6*-knockdown ONS76 cells. E: Western blotting detection of GPC6 and epithelial-mesenchymal transition (EMT) markers in DAOYs transfected with *GPC6* siRNA. F: Quantification of E-cadherin, N-cadherin, and MMP3 protein levels in (E). Data are presented as mean ± standard error of the mean (SEM) from three independent experiments. G and H: Transwell migration (G) and invasion assays (H) of *GPC6*-knockdown D283 cells. Quantification data of the transwell assay are shown as mean ± SEM from three independent experiments. Scale bars of panels A, B, C, D, G, and H, 200 μm. Data were analyzed using two-way ANOVA, and differences between groups were analyzed using Student's *t*-test. $^{**}P < 0.01$ and $^{***}P < 0.001$ by one-way ANOVA followed by Dunnett's test compared with the siNC group (A–D, G, and H) and unpaired *t*-test (F).

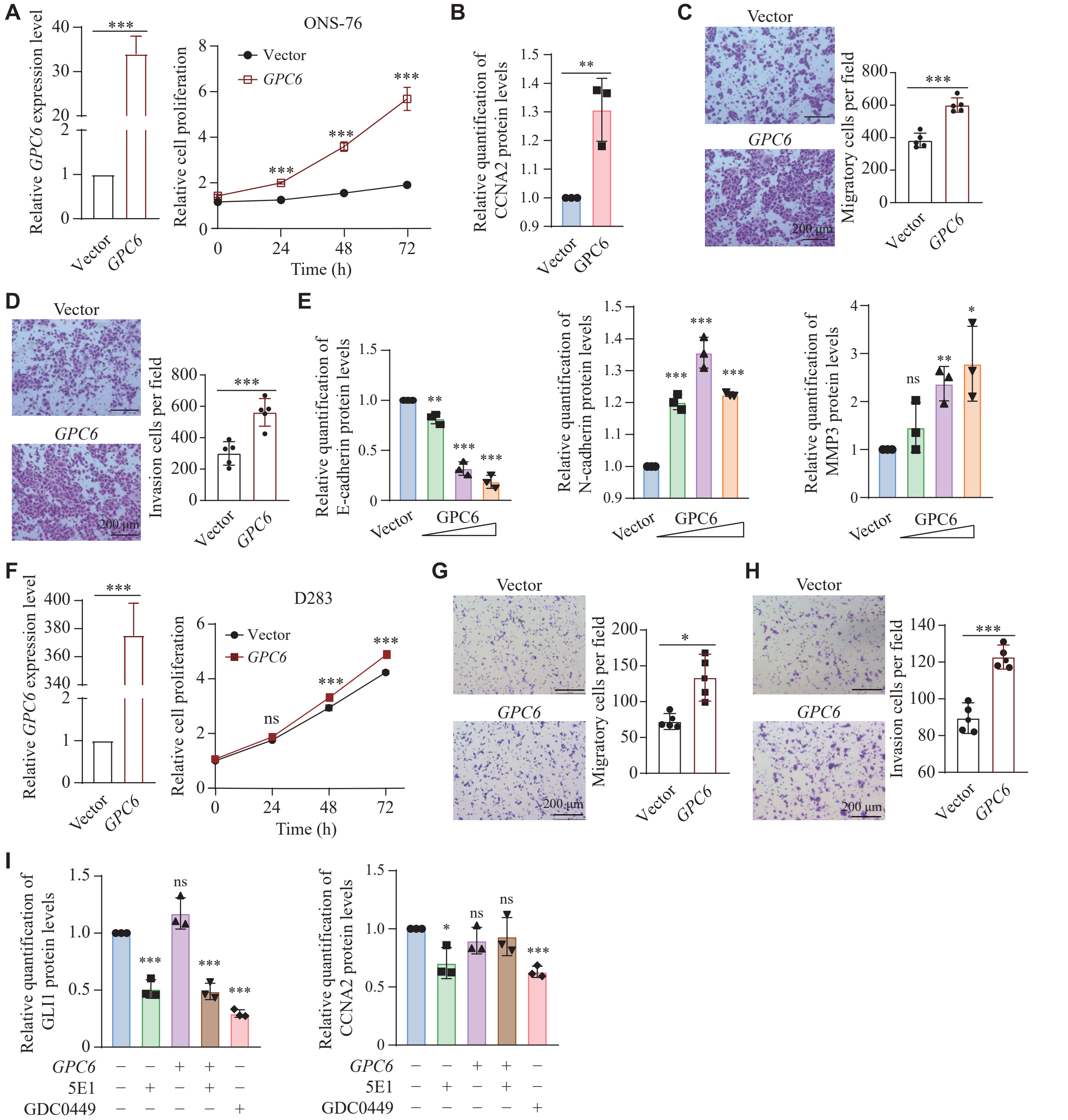

Supplementary Fig. 4 **GPC6 enhanced the proliferation, migration, and invasion of medulloblastoma cells.** A: CCK-8 assay of ONS-76 cells transfected with *GPC6* plasmids. B: Quantification of CCNA2 protein levels of ***Fig. 4C***. C and D: Transwell migration (C) and invasion assays (D) of *GPC6*-transfected ONS-76 cells. E: Quantification of E-cadherin, N-cadherin, and MMP3 protein levels of ***Fig. 4F***. F: CCK-8 assay of *GPC6*-transfected D283 cells. G and H: Transwell migration (G) and invasion assays (H) of *GPC6*-transfected D283 cells. I: Quantification of GLI1 and CCNA2 protein levels in ***Fig. 4L***. Western blotting detection of *GPC6*-transfected cells, with SHH-neutralizing antibody 5E1 (1∶500) or GDC0449 (100 μmol/L) for 24 h. Quantification data are shown as mean ± standard error of the mean from three independent experiments. *P < 0.05, **P < 0.01, and ns represents not significant, by unpaired t-test (reverse transcription-quantitative PCR data from panels A and F, B–D, G, and H), one-way ANOVA followed by Dunnett's test compared with Vector group (E and I), and two-way ANOVA followed by Dunnett's test compared with Vector group (CCK-8 data from A and F).

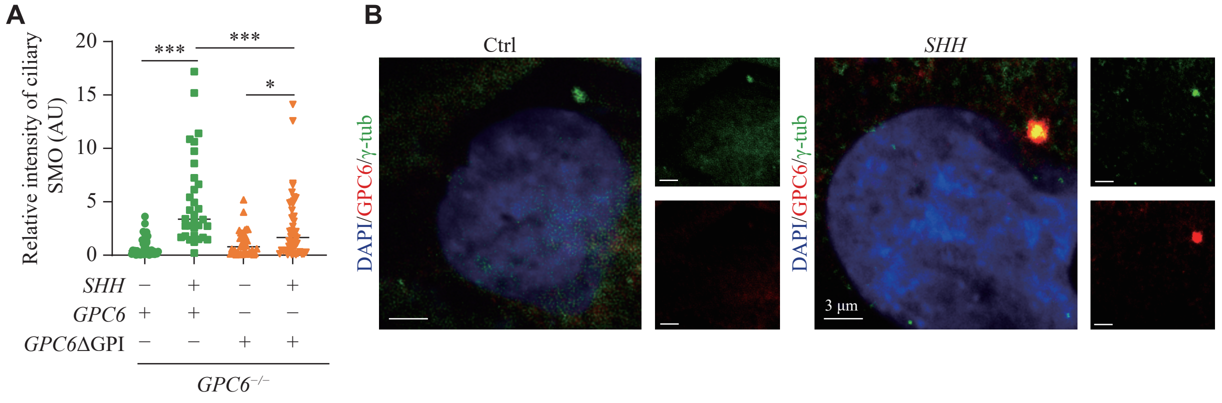

Supplementary Fig. 5 **GPC6 localized at the basal body of primary cilia.** A: Quantification of GPC6 and GPC6ΔGPI localization at the basal body of primary cilia following *SHH* stimulation ($n \geqslant 30$ in each group) of ***Fig. 4K***. Quantification data are shown as mean ± standard error of the mean (SEM) from three independent experiments. B: Immunofluorescence staining showing co-localization of GPC6 (red) and γ-tubulin (green) at the basal body in DAOY cells after *SHH* stimulation. Scale bar, 3 μm. $^{*}P < 0.05$ and $^{***}P < 0.001$ by one-way ANOVA followed by Tukey's multiple comparisons test (A).
